# The temporal dynamics of antimicrobial-resistant *Salmonella enterica* and predominant serovars in China

**DOI:** 10.1093/nsr/nwac269

**Published:** 2022-11-29

**Authors:** Yanan Wang, Yue Liu, Na Lyu, Zhiyuan Li, Sufang Ma, Demin Cao, Yuanlong Pan, Yongfei Hu, Hua Huang, George F Gao, Xuebin Xu, the Bacterium-learning Union, Baoli Zhu

**Affiliations:** International Joint Research Center of National Animal Immunology, College of Veterinary Medicine, Henan Agricultural University, Zhengzhou 450046, China; CAS Key Laboratory of Pathogen Microbiology and Immunology, Institute of Microbiology, Chinese Academy of Sciences, Beijing 100101, China; Department of Microbiology, Shanghai Municipal Center for Disease Control and Prevention, Shanghai 200336, China; CAS Key Laboratory of Pathogen Microbiology and Immunology, Institute of Microbiology, Chinese Academy of Sciences, Beijing 100101, China; CAS Key Laboratory of Pathogen Microbiology and Immunology, Institute of Microbiology, Chinese Academy of Sciences, Beijing 100101, China; CAS Key Laboratory of Pathogen Microbiology and Immunology, Institute of Microbiology, Chinese Academy of Sciences, Beijing 100101, China; CAS Key Laboratory of Pathogen Microbiology and Immunology, Institute of Microbiology, Chinese Academy of Sciences, Beijing 100101, China; Savaid Medical School, University of Chinese Academy of Sciences, Beijing 100049, China; CAS Key Laboratory of Pathogen Microbiology and Immunology, Institute of Microbiology, Chinese Academy of Sciences, Beijing 100101, China; State Key Laboratory of Animal Nutrition, College of Animal Science and Technology, China Agricultural University, Beijing 100193, China; Beijing Products Quality Supervision and Inspection Institute, Beijing 101300, China; CAS Key Laboratory of Pathogen Microbiology and Immunology, Institute of Microbiology, Chinese Academy of Sciences, Beijing 100101, China; Savaid Medical School, University of Chinese Academy of Sciences, Beijing 100049, China; Chinese Center for Disease Control and Prevention (China CDC), Beijing 102206, China; Department of Microbiology, Shanghai Municipal Center for Disease Control and Prevention, Shanghai 200336, China; CAS Key Laboratory of Pathogen Microbiology and Immunology, Institute of Microbiology, Chinese Academy of Sciences, Beijing 100101, China; Savaid Medical School, University of Chinese Academy of Sciences, Beijing 100049, China; Beijing Key Laboratory of Antimicrobial Resistance and Pathogen Genomics, Beijing 100101, China; Department of Pathogenic Biology, School of Basic Medical Sciences, Southwest Medical University, Luzhou 646000, China

**Keywords:** *salmonella enterica*, food safety, antimicrobial resistance, whole-genome sequencing, public health

## Abstract

*Salmonella enterica* is one of the most common bacterial pathogens in humans and animals. Systematic studies on the trends and geographical distribution of antimicrobial-resistant *Salmonella* and dominant serovars have been well studied in European and American countries while not in China. Here, taking the One-Health strategy, we used >35 000 *Salmonella enterica* isolates to explore the temporal and spatial dynamics of dominant serovars in China. We found that *Salmonella* Typhimurium was the dominant serovar causing human infection in China, which was consistent with Australia but inconsistent with North American and European countries. The proportion of *Salmonella* serovars Typhimurium, London, Rissen, Corvallis, Meleagridis, Kentucky, and Goldcoast showed an increasing trend during 2006–2019. We randomly selected 1962 isolates for comparative genomics and antimicrobial resistance studies and found that the number of antibiotic resistance genes (ARGs) per isolate increased 1.84 and 2.69 times of human and non-human origins, respectively, spanning 14 years. The proportion of antimicrobial-resistant *Salmonella* isolates had an increasing trend during 2006–2019, especially beta-lactam, quinolone, tetracycline, and rifampicin resistance. Moreover, we found that higher diversity of sequence types (STs) in *S*. Typhimurium than in other serovars, ST34 from pig and ST19 from chicken origin, were mainly associated with isolates causing child and adult gastro-infection, respectively. Our results fill in the data gap on the trends of dominant serovars and antimicrobial resistance of *Salmonella enterica* in China. These data provide useful information for public health decision-makers prioritizing interventions for foodborne diseases and food safety.

## INTRODUCTION

*Salmonella enterica* is a major cause of global foodborne diseases, and antimicrobial-resistant *Salmonella enterica* isolates pose a serious threat to public health [1]. *Salmonella enterica* consists of eleven subspecies [2]. *Salmonella enterica* subspecies *enterica* serovars Typhi (*S*. Typhi) and Paratyphi (*S*. Paratyphi) A, B, or C can cause typhoid and paratyphoid fevers in humans, collectively referred to as enteric fever [3], whereas other serovars are loosely described as non-typhoidal *Salmonella* (NTS). It was estimated that 14.3 million and 53.5 thousand cases of enteric fever and NTS invasive disease occurred in 2017, respectively, [4,5]. *Salmonella enterica* serovar Typhimurium (*S*. Typhimurium) was the most prevalent serovar causing human infection in Africa [6] and Australia [7]. The *S*. Enteritidis was the dominant serovar causing human infections in the United States [8] and European countries [9]. Recently, the World Health Organization (WHO) reported a multi-country outbreak of multidrug-resistant monophasic *S*. Typhimurium (*S*. I 1,4, [5],12: i:—ST34) infection linked to chocolate products (https://www.who.int/emergencies/disease-outbreak-news/item/2022-DON369), suggesting food vehicles associated with the transmission of this microbe. Although some studies reported that *S*. Typhimurium, *S*. Enteritidis, and *S*. I 1,4, [5],12: i:— were the most common serovars found among patients with diarrhea in China [10,11], most studies are from a single site, are of limited sample size, or of limited source and time.

Our previous study showed that a total of 322 serovars have been detected in China from 1951 to 2007 [12]. In recent years, some new serovars such as *S*. Telkebier [13], *S*. Uzaramo [14], and *S*. Changwanni [15] have been detected in China by our colleagues, and all of them were identified and reported for the first time in foreign countries. Although the prevalence and diversity of *Salmonella enterica* serovars from the five continents (Africa, the Americas, Asia, Europe and Oceania) have been well summarized in a previous meta-analysis [16], there is currently no systematic study of the epidemiology, temporal and spatial dynamics of dominant serovars of *Salmonella* in China. To fill this data gap, we have systematically analyzed the features of >35 000 *S. enterica* strains isolated from human and non-human origins enrolled in the Chinese Local Surveillance System for *Salmonella* database (also called Bacterium-learning Union) across 23 provinces or municipal cities in China over 14 years (from 2006 to 2019), which can support basic research and public health risk forecasts for *S. enterica*.

The emergence and spread of antimicrobial resistance (AMR) in *Enterobacteriaceae* pose a serious threat to human and animal health [17,18]. Notably, fluoroquinolone-resistant *Salmonella* spp. and extended-spectrum-beta-lactamase (ESBL)–producing *Enterobacteriaceae* have been listed by the WHO in 2017 as among the high priority pathogens posing a risk to human health and need for the research and development of new antibiotics [17]. It is estimated that a continued rise in AMR by 2050 will lead to 10 million people dying every year and a reduction of 2–3.5% in Gross Domestic Product [19].

Recently, whole-genome sequencing (WGS) has provided an unparalleled, powerful tool for understanding population dynamics, genomic epidemiology, and the investigation of outbreak dynamics, as well as the rapid temporal and spatial evolution of AMR in bacterial pathogens [20–23]. WGS-based predictions of AMR and serovar were highly consistent with the phenotypic determination results [24–27]. Moreover, comparative genomics could be performed to investigate the virulence profiles and mobile genetic elements (MGEs) of these isolates and their relationship with ARGs [28]. Therefore, to explore the trends and prevalence of AMR, as well as the distinct features of isolates of human and non-human origin, we sequenced and analyzed 1962 *S. enterica* isolates (involving 130 serovars) across 22 provinces or municipal cities in China. These analyses are intended to provide useful information for the maintenance of national biosecurity and food safety interventions, especially for monitoring and preventing the importation and spread of AMR and *S. enterica* in the population.

## RESULTS

### Geographical distribution of 35 382 *S. enterica* isolates in China

**Figure 1.**
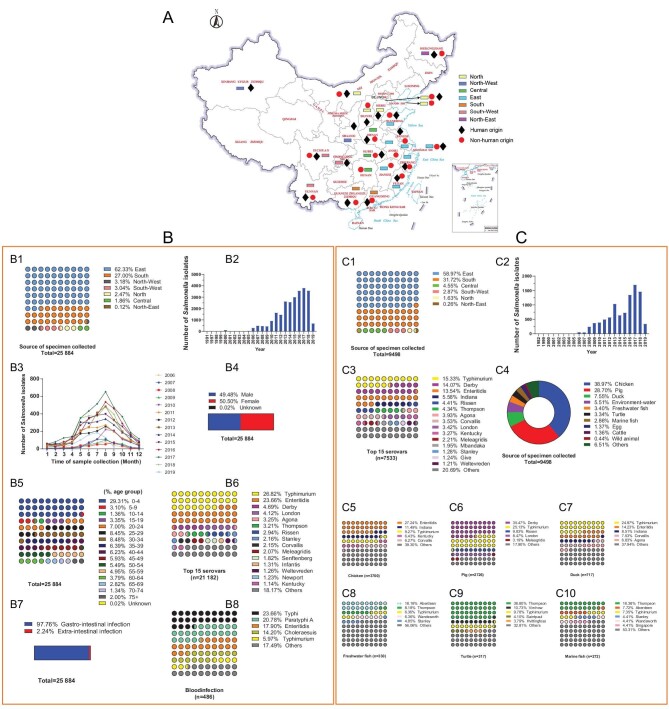
Geographical distribution and summary of 35 382 *S. enterica* isolates in China. A, Distribution of the 35 382 *S. enterica* isolates enrolled in the Bacterium-learning Union in 23 Chinese provinces. A total of 35 107 isolates (human, *n* = 25 635; non-human, *n* = 9472) collected between 2006 and 2019 and 275 isolates (human, *n* = 249; non-human, *n* = 26) collected between 1982 and 2005. To better analyze the geographical distribution of *S. enterica* among humans and non-humans in China, 23 provinces were divided into seven different regions on the basis of geographic proximity: North (Inner Mongolia, Beijing, Tianjin, Hebei, Shanxi), North-East (Heilongjiang), North-West (Shaanxi, Xinjiang), Central (Henan, Hubei), East (Shandong, Anhui, Jiangsu, Shanghai, Zhejiang, Fujian, Jiangxi), South (Guangdong, Guangxi), and South-West (Sichuan, Chongqing, Yunnan). All isolates were maintained at the Shanghai Municipal Center for Disease Control and Prevention for *Salmonella*. B, Summary of *S. enterica* strains isolated from human origin samples. B1, Collection region. B2, Collection year (1991–2019). B3, Time of sample collection. B4, Gender group. B5, Age group. B6, The proportions of fifteen dominant *Salmonella* serovars in China. B7, Infection type. B8, Isolates of bloodstream infection origin. Of the 25 884 isolates from humans (a total of 144 serovars), East and South contributed the largest numbers of isolates (both >25%), and North-East contributed the smallest (Fig. 1B1), which were collected between 1991 and 2019 throughout the year (Fig. 1B2), a higher number of cases was observed between May and September (Fig. 1B3), and the ratio of males to females in this study was 1 : 1 (Fig. 1B4). The age of subjects ranged from 1 day to 90 years and 32.41% were between 0–9 years old (Fig. 1B5). *S*. Typhimurium, *S*. Enteritidis, and *S*. Derby were the three most dominant serovars, accounting for 26.62%, 23.65% and 4.69%, respectively (Fig. 1B6); 97.76% of the 25 884 strains were isolated from gastrointestinal samples (Fig. 1B7). However, the main source of extra-intestinal was bloodstream infection, *S*. Typhi (115/486) was the most common among isolates from bloodstream infection samples, followed by *S*. Paratyphi A (101/486) (Fig. 1B8). C, Summary of *S. enterica* strains isolated from non-human origin samples. C1, Collection region. C2, Collection year (1982–2019). C3, The proportions of fifteen dominant *Salmonella* serovars in China. C4, Source. C5, Chicken. C6, Pig. C7, Duck. C8, Isolates of bloodstream infection origin. Freshwater fish. C9, Turtle. C10, Marine. Of the 9498 isolates of non-human origin (a total of 120 serovars), *S*. Typhimurium was the most dominant serovar (Fig. 1C3), 87.54% (8315/9498) were isolated from food (Fig. 1C4) of animal origin (mainly including chicken, pig, duck, freshwater fishes, turtles, marine and cattle), and the distribution of serovars varied among different sources (Fig. 1C5-C10).

From 1 September 1982, to 28 September 2019, 35 382 serotyped *Salmonella* spp. isolates (164 serovars) were registered at the Bacterium-learning Union (Fig. 1). The large collections were derived from 23 provinces or municipal cities in China (Fig. 1A). Of the 35 382 isolates, 34 804 (98.37%) belong to NTS, 419 (1.18%) to *S*. Paratyphi, 159 (0.45%) to *S*. Typhi (Fig. S1). A detailed description of the isolates is summarized in Fig. 1B and C. *S*. Typhimurium, *S*. Enteritidis, and *S*. Derby were the three most dominant serovars, accounting for 26.62%, 23.65% and 4.69%, respectively, (Fig. 1B). However, *S*. Typhi (115/486) was the most common among isolates from bloodstream infection samples, followed by *S*. Paratyphi A (101/486) (Fig. 1B). Overall, *S*. Typhimurium (23.73%, 8397/35 382) was the most common serovar in both human and non-human origins, followed by *S*. Enteritidis, *S*. Derby, *S*. London and *S*. Thompson (Fig. S2A). Results from the isolated *S. enterica* strains’ serotyping show that in South and South-West regions, *S*. Typhimurium was the major serovar; in East, North, and North-West regions, *S*. Enteritidis was the major serovar (Fig. S2B & C).

Moreover, we observed key differences between the distribution of Salmonellosis in China and other countries and regions. The most common serovar causing human infection was similar to Australia while inconsistent with the USA and England. In contrast to these countries, 4 (Meleagridis, London, Corvallis, Rissen) of the 10 most common serovars identified in our database did not appear on their lists (Table S1).

### Dominant subtype switch in *S. enterica* serovars during 2006–2019 in China

**Figure 2.**
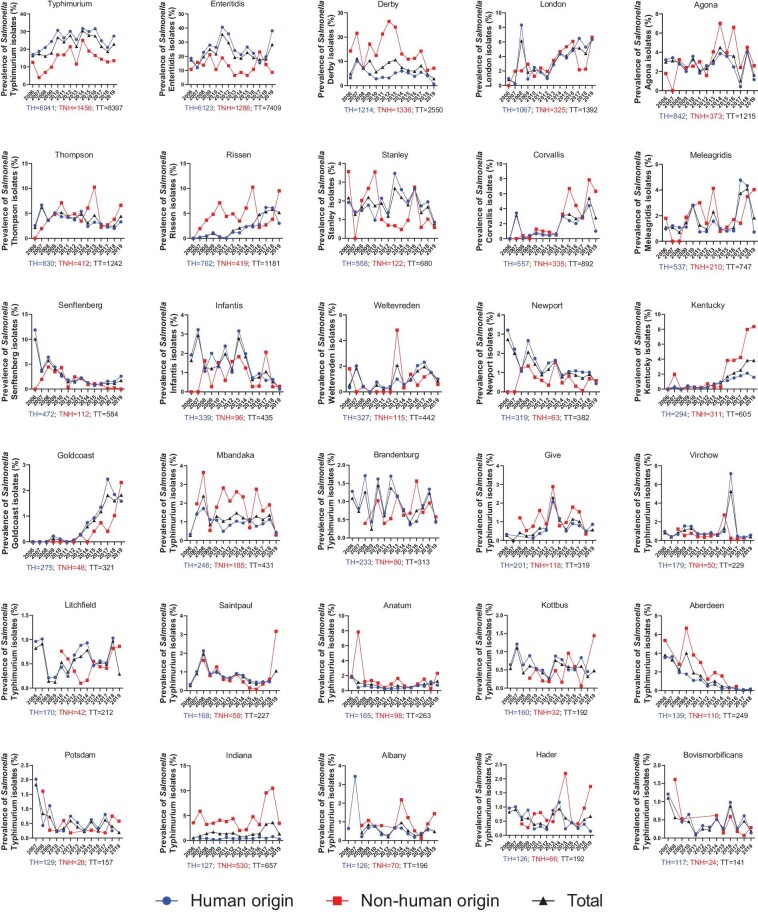
Temporal trends of *S. enterica* serovars during 2006–2019 in China (top 30 serovars). TH: The total number of *Salmonella* isolates of human origin. TNH: The total number of *Salmonella* isolates of non-human origin. TT: The total number of *Salmonella* isolates of non-human origin.

To better understand the temporal dynamics of *S. enterica* serovars in China, a detailed description of the top 30 NTS serovars is summarized in Fig. 2. Generally, the proportion of *Salmonella* serovars Typhimurium, London, Rissen, Corvallis, Meleagridis, Kentucky, and Goldcoast had an increasing trend during 2006–2019; while the proportion of *Salmonella* serovars Derby, Senftenberg, Infantis, Newport, Aberdeen, Potsdam, Bovismorbificans, showed a downward trend (Fig. 2). *S*. Enteritidis was the most common serovar causing human infection in China in 2006 (18.59%) and 2012 (35.90%), while *S*. Typhimurium became the most common serovar from 2013 to 2018, however, *S*. Enteritidis became the dominant serovar again in 2019 (Fig. 2). The prevalence of *Salmonella* serovars Derby, Rissen, Kentucky, Corvallis, Mbandaka, Aberdeen, and Indiana from non-human origin was higher than that of human origin. Moreover, the percentage of *S*. Typhi isolates that cause typhoid fever has been decreasing in recent years (Fig. S3). Notably, an average of 62 and 46 serovars are detected annually in *S. enterica* isolates of human and non-human origin, respectively. The number of serovars detected had an increasing trend with fluctuation over time during 2006–2019 (Fig. S4A), and new serovars can be detected annually (Fig. S4B).

### Serotyping and multi-locus sequence type based on WGS data

To understand the genomic characteristics and AMR trends of *S. enterica* isolates, we performed Illumina sequencing on 1962 isolates from 22 Chinese provinces or municipal cities (Figs S5 and S6 and Table S2). The isolate collection represented a wide variety of isolation sources involving five subspecies of *S. enterica* and a large time span, ranging in date from 1982 to 2019 (Fig. S5A–C). Among the 1962 *S. enterica* isolates, a total of 130 different serovars were included, *S*. Typhimurium was the most common serovar, accounting for 25.59% (Table S3 and Fig. S5D). Notably, 29 *S. enterica* serovars have not been isolated and identified in China before this study, the genomic characteristics of these strains were summarized (Table S4).

Based on WGS data, all 1962 isolates were well serotyped by both traditional serology and WGS using a combination of SeqSero2 and SISTR tool for serovar prediction (100%) (Fig. S5E and Table S5). It should be noted that biphasic *S*. Typhimurium (*S*. 1,4, [5] : 12 :  i : 1,2) and monophasic *S*. Typhimurium (*S*. I 1,4, [5],12: i:—) were not well distinguished in our database, 164 *S*. I 1,4, [5],12: i:— strains were identified using WGS prediction, accounting for 32.67% of the 502 strains (Table S5). A detailed description of *S*. 1,4, [5] : 12 : i : 1,2 and *S*. I 1,4, [5],12: i:— strains from humans, pigs, and chickens are summarized in Fig. S7A–C. Among these isolates of human origin, the proportion of *S*. I 1,4, [5],12 : i :— have shown an increased trend since 2011, while exceeding the proportion of *S*. 1,4, [5]:12: i:1,2 in 2016 (Fig. S7D). Notably, of the 79 *S*. I 1,4, [5],12: i:—isolates of human origin, more than 63.29% (50/79) were recovered from children aged ≤5 years (Fig. S7E and F).

In addition to serotyping, multilocus sequence typing (MLST) is an accurate, robust, reliable, high throughput typing method that was also used as an unambiguous tool to examine bacterial relatedness [29–31]. We found that 200 different STs among the 1962 isolates and 12 previously unknown STs were assigned in seven serovars (16 isolates) (Fig. S8 and Table S6), suggesting that well-known and previously unidentified ST groups are both co-existing and evolving. Generally, ST11 was the dominant ST in the 1962 genomes, followed by ST34 and ST19 (Fig. S9A). Moreover, the diversity of STs has had an increasing trend over time in recent years (Fig. S9B). Among the 1159 isolates of human origin, ST34 was the dominant ST in six Chinese areas, except for North-West (Fig. S9C). Of the 803 isolates of human origin, ST34 was the dominant ST in the South, South-West, Central, and North, while ST11 was the dominant ST in the East (Fig. S9C).

### Temporal changes of AMR, ARGs and virulence factors

A total of 118 different AMR genotype patterns involving 12 antimicrobials were found in the 1958 isolates (Table S7). Among these beta-lactamase–producing isolates, 59.98% of these were the CTX-M-type beta-lactamases. Overall, 13 *bla*_CTX-M_ genes were identified. While the most prevalent beta-lactamases were *bla*_TEM-1B_ (*n* = 547, Table S8). Genes conferring resistance to sulphonamides (40.57%, 796/1962), beta-lactams (39.50%, 775/1962), tetracyclines (34.86%, 684/1962) and phenicols (22.27%, 437/1962) were frequent in the present collection. Worryingly, 205 and 1537 of 1962 isolates were found carrying the plasmid-mediated quinolone resistance genes (PMQRs) (Table S9) and genomic mutations in the quinolone resistance-determining regions (QRDRs) (Table S10), respectively. Although few PMQRs (1.09%, 4/366) were mainly identified in *S*. Enteritidis strains, a higher prevalence of genomic mutations in the QRDRs (97.54%, 357/366) was observed. The prevalence of genomic mutations in the QRDRs in *S*. Enteritidis strains was higher than in mobile beta-lactam resistance genes.

We found that 16, 2, and 9 isolates were positive for the mobile colistin resistance genes *mcr-1, mcr-3*, and *mcr-9*, respectively (Table S11). Interestingly, 9 of 16 *mcr-1* carrying isolates belonged to *S*. I 1,4, [5],12: i:—ST34. It was noteworthy that *fosA3* and *fosA7* which were contributing to reduced fosfomycin susceptibility were identified, including *fosA7* (4.33%, 85/1962) and *fosA3* (1.53%, 30/1962) (Table S12). In addition, the mobile tigecycline resistance gene *tet*(X4) was identified in an *S*. Llandoff ST8300 isolate (0.05%, 1/1962) from a human stool sample in 2016.

**Figure 3.**
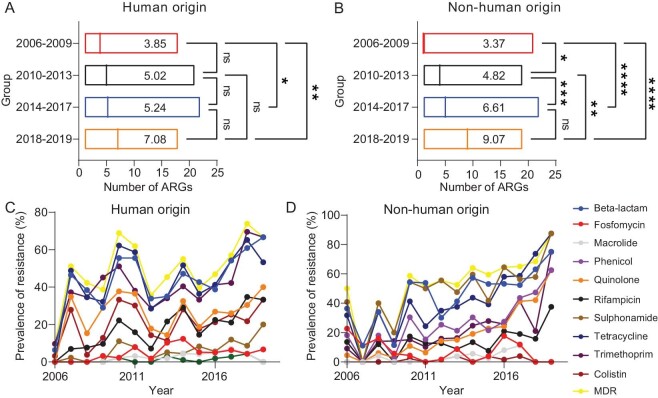
Temporal and spatial changes in AMR of NTS in China. A and B, The distribution of numbers of ARGs in each NTS isolate from both human origin (A) and non-human origin (B) grouped by sampling time. (Both, *n* = 1713; Human origin, *n* = 937; Non-human origin, *n* = 776). The mean ARG count for each period is noted. Multiple comparisons were performed by the Kruskal–Wallis test using GraphPad Prism version 8.0. The ‘*’ on the right represent *P*-values. *: *P* < 0.05; **: *P* < 0.01; ***: *P* < 0.001. ns, no significant difference. C, Temporal dynamic of AMR genotypes in NTS isolates from human origin (*n* = 937). D, Temporal dynamic of AMR genotypes in NTS isolates from non-human origin (*n* = 776).

Between 2006 and 2019, the average number of ARGs carried by NTS genomes increased in both human and non-human origins (Fig. 3A and B), and increased trends were also observed in 4 of 12 antimicrobials of human origin and 7 of 12 of non-human origin (Fig. 3C and D). In particular, the prevalence of beta-lactam resistance genes from 25.93% in 2006–2009 increased to 68.71% in 2014–2017. In general, the proportion of AMR isolates, including beta-lactam, quinolone, tetracycline, and rifampicin which are key antimicrobial agents in human and/or veterinary medicine, have an increased trend over time in both human and non-human origins. The proportion of isolates conferring resistance to phenicol, sulphonamide, and trimethoprim showed a significantly increased trend in non-human origin, while the proportion of sulphonamide- and trimethoprim-resistant strains fluctuated and increased in human origin. This phenomenon may be explained by the use of antibiotics (for example, broad-spectrum penicillins, cephalosporins, fluoroquinolones, macrolides, and trimethoprim) in China during the same period, the data of antibiotic use was obtained from the ResistanceMap (https://resistancemap.cddep.org/CountryPage.php?countryId=70&country=China+).

The prevalence of MDR *Salmonella* isolates across different areas was distinct (Table S13). Chicken and pig are the main sources of non-human origin isolates, the proportion of MDR rates also showed an increased trend in different sampling periods (Fig. S10). Positive correlations with resistance to multi-drugs were observed with some serovars. In total, 55.28% (429/776) and 49.29% (452/917) of non-human and human origin NTS isolates had an MDR gene type which was distributed in 39 and 46 serovars, respectively. Among these serovars, *Salmonella* I 1,4, [5],12: i:— and Choleraesuis exhibited a relatively high proportion of MDR gene types, both exceeding 92.0% (Table S14). However, the proportion of MDR rate in *S*. Goldcoast of human origin (66.67%) is higher than that of non-human origin (41.67%).

**Figure 4.**
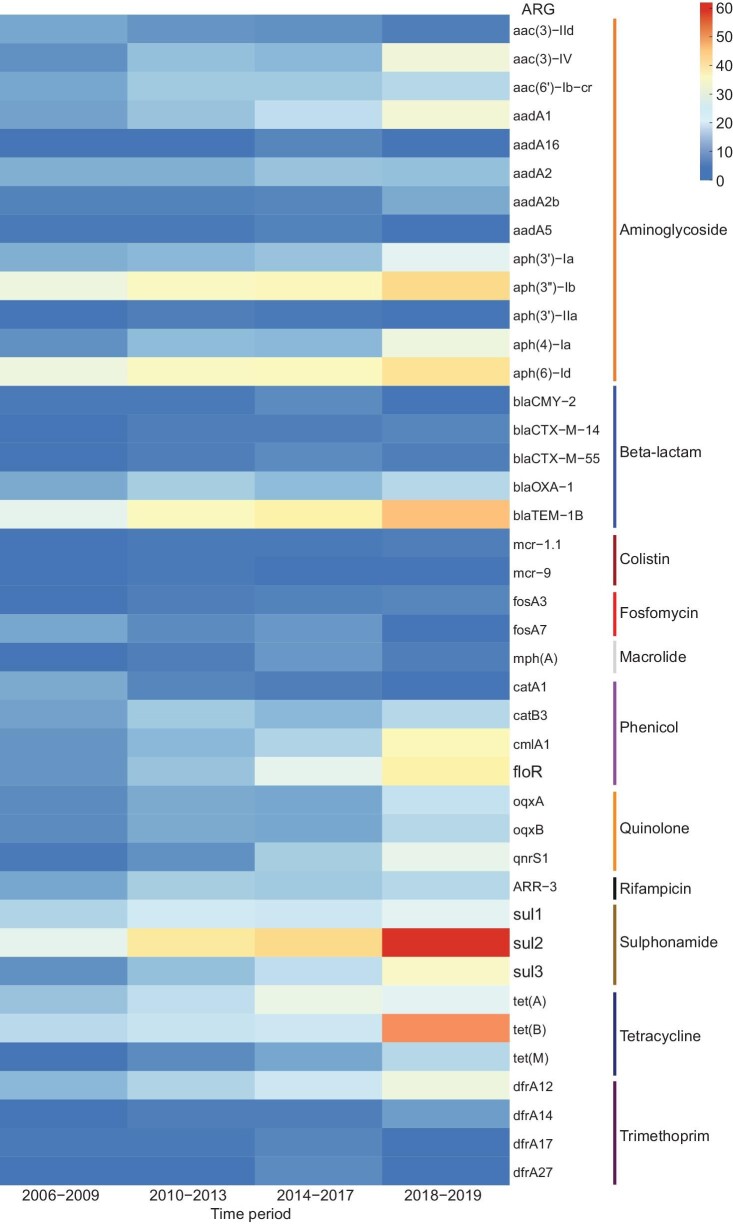
Temporal changes in the prevalence of ARGs among NTS isolates. Only significant temporal trends and critically important resistance genes are shown (1% significance level). Colored bars represent the predicted phenotype conferred by ARGs, and they are clustered by their resistance to different classes of antibiotics. ARGs that were detected at >1% prevalence in NTS genomes are shown (*n* = 1710).

The increasing dynamics of individual ARGs in different sampling periods were also observed in beta-lactam ARGs *bla*_TEM-1B_, *bla*_OXA-1_, and *bla*_CTX-M-14_, aminoglycoside ARGs *aac(3)-IV, aac(6′)-Ib-cr, aadA1, aph(3′)-Ia, aph(3′′)-Ib, aph(4)-Ia*, and *aph(6)-Id*, fosfomycin ARG, *fosA3*, phenicol ARGs *catB3, cmlA1*, and *floR*, rifampicin ARG *ARR-3*, sulphonamide ARGs *sul2* and *sul3*, tetracycline ARGs *tet(B)* and *tet(M)*, and Trimethoprim ARGs *dfrA12* and *dfrA14* (Fig. 4). However, decreasing dynamics were observed in several ARGs, for example, *aac(3)-IId* and *catA1*.

For virulence-associated genes, a total of 129 different virulence factors (VFs) were found (Table S15). No obvious trends or changes in VFs carried by human and non-human origin isolates were observed. Noticeably, the typhoid toxin cdtB was observed in 37 different serovars (Table S16). Most virulence genes are located within highly conserved *Salmonella* Pathogenic Islands (SPIs) on the chromosome and are usually acquired by lateral gene transfer [32]. SPI-1 was the predominant SPI, identified in 99.80% of all *S. enterica* strains, followed by SPI-2, SPI-3, and SPI-5, accounting for 99.69%, 99.69%, and 99.64%, respectively (Table S17). *Salmonella* genomic island 1 (SGI1) was identified in 32 isolates (13 serovars) (Table S18). SGI1 is an integrative mobilizable element initially characterized in *S*. Typhimurium DT104 strains that play an important role in the capture and spread of MDR, subsequently it has been found in clinical isolates of *Salmonella* serovars and some members of the *Enterobacteriaceae* [33]. The *Yersinia* high pathogenicity island (HPI) encodes a yersiniabactin-mediated iron acquisition system present in highly pathogenic strains of *Yersinia* and several members of the *Enterobacteriaceae*, for example, *Escherichia coli, Citrobacter* spp., *Klebsiella* spp., *S. enterica* subspecies III and VI, and *S*. Senftenberg [34]. To our knowledge, HPI is rare in *S*. Typhimurium, and this is the first report of HPI in *S*. Typhimurium strains isolated from humans.

### Investigation of MGEs associated with the transfer of ARGs and VFs

Next, we searched for MGEs in isolates from different sources and geographical areas and, where possible, analyzed linkages between MGEs and the carriage of specific ARGs. In total, 7821 MGEs categorized into 111 MGE groups (Table S19) were predicted, of which the majority were either Insertion sequences (ISs, 75.96%) or Miniature Inverted Repeats transposable elements (MITEs, 18.72%). MITEEc1, IS*Ecl10*, IS*Sen1*, IS*Sty2*, IS*26*, and IS*Kpn2*, were the six most common types of integrating MGEs (iMGEs). Interestingly, we found that some ISs usually co-existed with specific ARGs, for example, IS*903B*-*mcr-9*-IS*26*, IS*Apl1*-*mcr-1*-PAP2, and TnAs2-*mcr-3*-dgkA-IS*kpn40* (Fig. S11). A total of 344 Tns were detected, Tn2 was the most common element (*n* = 190). Moreover, 24 different Composite transposons (ComTns) were detected in 208 isolates (Table S19).

In general, the number of MGEs per isolate was positively correlated with number of ARGs carried in isolates of both human (P < 0.0001, R^2^ = 0.4562; Fig. S12A) and non-human origin (P < 0.0001, R^2^ = 0.4628; Fig. S12B). However, no correlation was observed between the number of MGEs and VFs per isolate (Fig. S12C and D).

Although plasmids are abundant in *S. enterica* [35], with the hybrid assemblies and predictions combined, we found a high proportion (27.47%, 539/1962) across the entire collection of the *S. enterica* isolates to lack plasmid sequences. Notably, these isolates (72.53% of 1962) carried at least one plasmid replicon and were distributed in 97 serovars (Table S20); 85.70% (665/776) and 77.62% (725/934) of non-human and human origin isolates (Fig. S13). A total of 3679 plasmid replicons divided into 66 classes were found among these isolates, with a maximum of nine plasmid replicons observed in a single isolate. Plasmid replicons of IncFII(S), IncFIB(S), Col(pHAD28), IncHI2, and IncX1 were the five most common types of replicons, accounting for 28.49%, 27.42%, 16.82%, 13.05%, and 12.95% of the 1962 isolates, respectively. Further analysis suggested that specific serovars were contributors to the high plasmid carrier rates, particularly I 1,4, [5],12: i:—, 1,4, [5] : 12 : i : 1,2, Choleraesuis, Enteritidis, and Goldcoast.

We found that 67.99% (1334/1962) of the isolates contained at least one intact prophage in their genomes (Table S21). A total of 1976 prophages divided into 71 classes were found among the 1962 isolates, with a maximum of four intact prophages observed in a single isolate. Interestingly, *Gifsy 2, Salmon 118 970 sal3* and *Gifsy 1* were the three most common prophages (*n* = 537, 490 and 249, respectively).

### More ARGs, VFs, and MGEs were observed in *S. enterica* isolates of non-human origin

We subsequently explored the shared and distinct features of WGS genomes of human and non-human origin (Fig. S14). Overall, the NTS strains isolated from non-human origin possessed more ARGs, virulence genes, MGEs, and AMR resistance phenotype than that from human origin (Figs S15 and S16). Of these WGS isolates of non-human origin, the strains isolated from pigs harbored more ARGs and MDR genotypes than the others (Fig. S17). We also explored the shared and distinct features of WGS isolates from humans, wet markets, and the environment (Huangpu River water) in Shanghai, which is one of the largest cities in China. Among these serovars, ARGs, MGEs, and plasmids identified in strains of human origin, most of them could be found in strains isolated from wet markets, suggesting that AMR-*Salmonella* could circulate among human, food (wet markets) and the environment in Shanghai (Fig. S18).

Among these WGS isolates, 74.13% (212/287) and 90.30% (763/845) were from blood (Fig. S19A) and diarrhea stool samples (Fig. S19B), respectively. We found that more ARGs and fewer VFs in *S*. Enteritidis from gastrointestinal infection than that from extra-intestinal infection (Figs S20 and S21).

Moreover, a total of 550 and 8 strains of *S*. Kentucky and *S*. Chester were identified in our database, respectively (Figs S22 and S23). Of the 1962 WGS isolates, a total of 10 *S*. Kentucky strains were included, including five ST198 and ST314, respectively (Table S22). All five *S*. Kentucky ST198 isolates showed an MDR genotype, with three carrying *bla*_CTX-M-14b_ and two carrying *bla*_CTX-M-65_, respectively. One *S*. Kentucky ST198 had an MDR genotype first detected in human fecal samples in Shanxi province. Among the five WGS *S*. Chester strains, four and one belonged to ST343 and ST411, respectively. Notably, the *S*. Chester ST343 had been isolated from a foreigner's fecal sample on 16 April 2013 (Table S23).

### Phylogenetic analysis of *S. enterica* genomes

**Figure 5.**
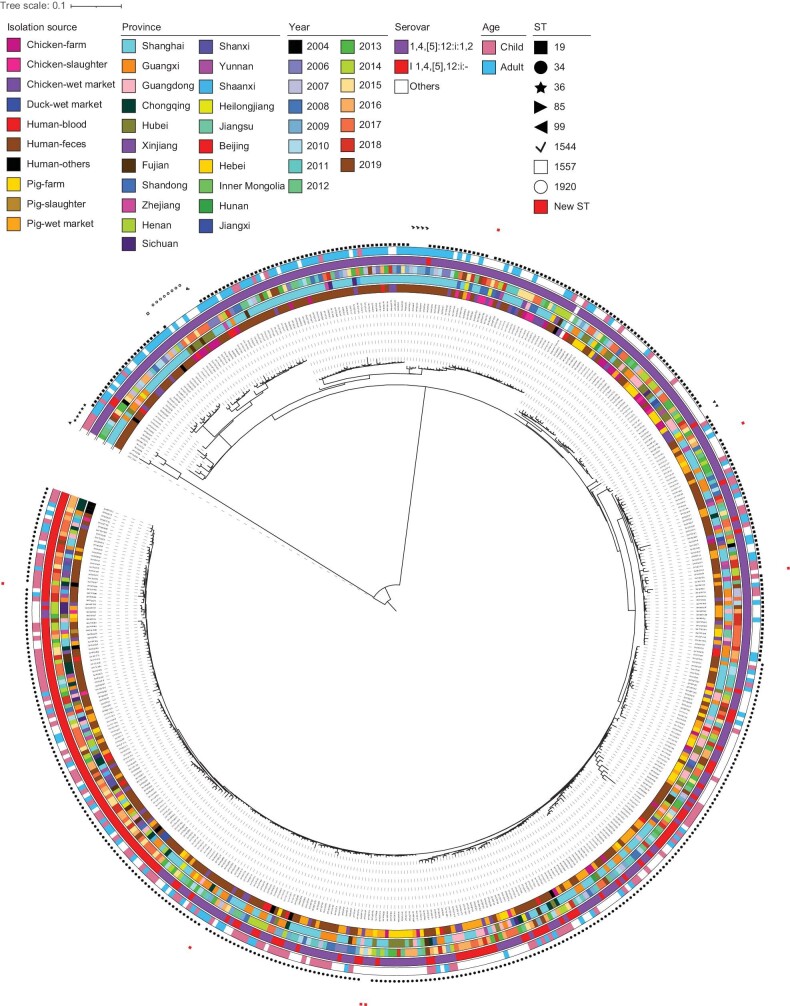
Phylogenetic analysis of *S*. Typhimurium and its variant I 1,4, [5],12: i:— isolates from humans and food animals in China. Maximum-likelihood tree of 502 *S*. Typhimurium and monophasic isolates was constructed by using 50 061 SNPs identified by reference to the complete genome sequence of *S*. Typhimurium strain LT2. The tree is rooted with *S*. Paratyphi A strain AKU1_12601 complete genome sequence as an outgroup. From the inner to outer circles are 1. Isolation source, 2. Province, 3. Year, 4. Serovar, 5. Age group, 6. ST of the isolates.

To determine the relationship between *S*. 1,4, [5] : 12 : i : 1,2 and I 1,4, [5],12: i:— isolated from humans and food animals in China, we constructed maximum-likelihood phylogeny of 502 strains (Fig. 5). Higher diversity of STs (including six novel STs, ST8333, ST9160, ST9161, ST9162, ST9163 and ST9164) in *S*. Typhimurium than other serovars, ST34 has replaced ST19 as the dominant ST in recent years. It is worth noting that ST34 from pigs and ST19 of chicken origin were mainly associated with isolates that caused children and adult infection, respectively (Fig. 5). We also performed phylogenomic analysis on the 338 *S*. 1,4, [5] : 12 : i : 1,2 (Fig. S24) and 164 *S.* I 1,4, [5],12: i:— (Fig. S25) isolates, respectively, to further explore their genetic relationship. The results showed that ST34 and ST19 isolates were divided into multiple clades, respectively.

**Figure 6.**
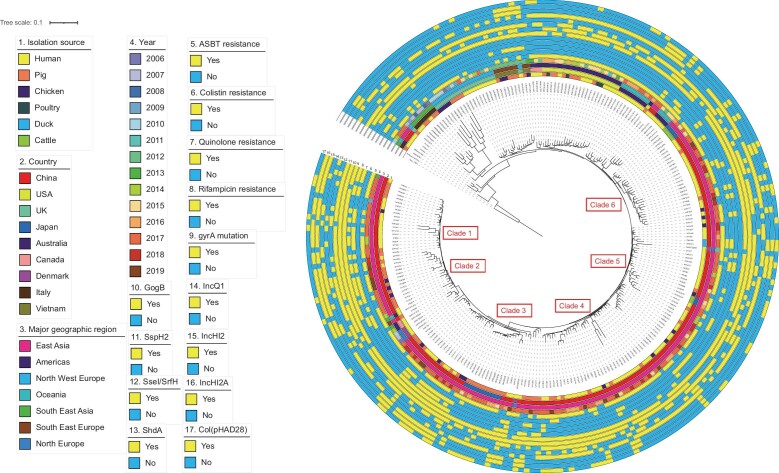
Phylogenetic analysis of global *S*. I 1,4, [5],12: i:—ST34 genomes. Maximum-likelihood phylogenetic tree based on the 157 genome sequences from this study and 96 publicly available *S*. I 1,4, [5],12: i:—ST34 isolates. The tree is rooted with *S*. Paratyphi A strain AKU1_12 601 complete genome sequence as an outgroup. The tree is based on 19 436 chromosomal SNPs. From the inner to outer circles are 1. Isolation source, 2. Country, 3. Major geographic region, 4. Year of the isolates, 5. ABST resistance (genes detected for aminoglycoside, beta-lactam, sulphonamides and tetracycline resistance), 6. Colistin resistance, 7. Quinolone resistance, 8. Rifampicin resistance, 9. gyrA mutation, 10. GogB, 11. SspH2, 12. SseI/SrfH, 13. ShdA, 14. IncQ1, 15. IncHI2, 16. IncHI2A, 17. Col(pHAD28). 5–8: Resistance phenotype. 10–13: Virulence factors. 14–17: replicon types. From the phylogenetic tree, we found that 1) clade 1 and 4 only included Chinese strains; 2) clade 2 mainly included Chinese strains and one Australian strain which were closely related to 2 and 3 strains from Canada and Denmark, respectively; 3) clade 3 were Chinese isolates and were clustered with those isolated from pigs in Japan; 4) clades 5 and 6 included Chinese strains and strains isolated from cattle from Japan. In addition, two Chinese isolates from humans clustered with one American isolate from pig and Australian strains from humans.

To further explore the genetic relationship of *S.* I 1,4, [5],12: i:—ST34 isolates collected from China and other geographical regions, we performed phylogenetic analysis of 157 Chinese isolates and 96 publicly available genomes from different sources, a random subset of isolates that was representative of lineages in recent reports [36,37] (Fig. 6). These results suggested that Chinese isolates were different from those in Southeast Asia, Northwest Europe, Southeast Europe and America, and with a local evolution. Previous studies reported that the global dissemination of the *S.* I 1,4, [5],12: i:— initially from Europe, then spread to Asia and the USA [36,38].

To investigate the phylogenomic relationship with our dataset of *S*. Enteritidis ST11 genomes, we generated a maximum-likelihood phylogenic tree inferred from the 6976 single nucleotide polymorphisms (SNPs). From the SNP phylogeny, we found that *S*. Enteritidis genomes caused human infections mainly associated with chicken, and the majority of *S*. Enteritidis carrying the *bla*_TEM-1B_ gene also showed the MDR pattern (Fig. S26). To understand the origins and genetic relationship of *S*. Choleraesuis, we performed a combined genomic analysis, contextualizing Chinese data with *S*. Choleraesuis isolates from Europe, Asia and the Americas. The phylogenetic tree shows that the Chinese strains isolated from humans or pigs were closely related to strains isolated from India, Thailand, the United Kingdom and Denmark, but distant from strains from Germany, Vietnam, Brazil and France (Fig. S27). Taken together, these findings suggested that *S*. Choleraesuis strains collected in this study have various geographic origins and evolved locally. We also explored the relationship between *S*. Heidelberg genomes recovered from humans and chickens in China and chickens imported from the United States and Brazil. The Chinese strains isolated from chickens were closely related to strains isolated from chickens imported from Brazil, distinct from the strain isolated from a human in China (Fig. S28). It should be noted that the result relies on a single *S*. Heidelberg isolate of humans for comparison.

To understand the population structure currently causing typhoid in China, we constructed a maximum-likelihood phylogeny of 64 strains of *S*. Typhi (Fig. S29). The results based on 8772 SNPs showed the division of isolates into two clades, and both of them were observed in Xinjiang. A phylogenetic analysis of Paratyphi B and its variants revealed that I 1, 4, [5], 12: b:— and *S*. Paratyphi B var. Java ST42 genomes recovered from humans clustered together (Fig. S30). Moreover, the isolates Paratyphi B ST86 and Paratyphi B var. Java ST42, ST43, ST423 from aquatic animals were clustered with humans. These results indicated that aquatic animals are potential host and transmission vectors of Paratyphi B ST86 and its variants.

## DISCUSSION

To our knowledge, this is the largest collection and first systematical study reporting the temporal and spatial dynamics of *S. enterica* serovars across 23 provinces or municipal cities over 14 years in China. *S*. Typhimurium (26.82%) is the most common serovar causing human infection in China (Fig. 1B), which was similar to Africa [6], Belgium [39], and Australia [7], and inconsistent with other high-income countries and regions [9,40]. *Salmonella* spp. was the second primary (6.8%) bacterial pathogen of foodborne disease outbreaks in China, many of which were linked to the consumption of animal source foods, which were considered to be the major reservoirs from which *Salmonella* was spread [41,42]. Moreover, our data also showed that *Salmonella* serovars Enteritidis, Derby, Typhimurium, Thompson, and Aberdeen were the most common serovars detected in chicken, pig, duck, aquatic product, and turtle, respectively, which is consistent with the findings in previous reports [42,43].

Among the 502 WGS *S*. Typhimurium strains registered at the Bacterium-learning Union, 32.67% of strains (*n* = 164) were predicted as *S*. I 1,4, [5],12: i:—, indicating WGS can help us better distinguish *S*. 1,4, [5] : 12 : i : 1,2 and *S*. I 1,4, [5],12: i:—. Previous studies have shown that incongruent matches were present between conventional serotyping and WGS identification using SeqSero2 or SISTR in *S. enterica* strains (for example, serovar variants) from foods and human beings [27,44–46]. However, these problems could partly be overcome by using WGS in combination with *in silico* analysis tools. Notably, a total of 29 serovars that have not previously been reported in China were identified by conventional serotyping and WGS prediction in this study. Briefly, this is the largest collection of conventional serotyping results by using WGS prediction *Salmonella* serovars (a total of 130 serovars) in China. A total of 164 serovars were identified in China in this study, however with the rapid development of the economy and global trade, and the aging of human beings, the risk of foodborne pathogens’ dissemination will further increase, and more and more *Salmonella* serovars will be detected in China.

Phylogenetic analysis revealed that ST34 from pig and ST19 from chicken origin were mainly associated with isolates causing child and adult gastro-infections, respectively. The previous study reported by the CDC and EFSA also emphasized that pig and pork meat are the main sources of *S*. Typhimurium and its variant [16]. Significant differences were observed in Chinese *S*. I 1,4, [5],12: i:—ST34 strains than strains in Southeast Asia, Northwest Europe, Southeast Europe and America. These results showed that a local evolution was present in Chinese strains.

For most drugs, the detection of resistance genes was both sensitive (>98%) and specific (>99%) in predicting AMR phenotype [24–27]. WGS revealed a diversity of known genetic mechanisms for AMR that accounted for >45% of isolates that had an MDR genotype. From 2006 to 2019, the proportion of AMR *S. enterica* strains showed an increasing trend in both human and non-human origin (Fig. 3C and D). Moreover, we also found the AMR rate of most antimicrobials of human origin isolates decreased in 2012, which was probably influenced by the policy for antimicrobial management (http://www.gov.cn/flfg/2012–05/08/content_2132174.htm). A recent study reported that the prevalence of resistance in *Salmonella* showed an increased trend after 2010 in penicillin in chicken, and in sulfonamide, penicillins, and tetracycline in pigs [22]. A similar landscape was also observed in other countries, for example, the prevalence of MDR was generally low ranging from 3.4% in 1984 to a high of 9.7% in 2013 in Australia [7]. Although some changes in antimicrobial usage policy in China during this timeframe, including human medicine [47] and animal husbandry (http://www.gov.cn/gongbao/content/2013/content_2528114.htm, http://www.moa.gov.cn/govpublic/ncpzlaq/201507/t20150723_4759838.htm), it is also easy to obtain antimicrobials without a prescription in retail pharmacies [48]. In some serovars, the prevalence of MDR was greater than 50%, while the serovars were different from that in Australia [7]. In addition, more AMR genes and MGEs were identified in strains such as *S*. 1,4, [5] : 12 : i : 1,2 and *S*. I 1,4, [5],12: i:— isolated from non-human rather than human origin, suggested a possible transmission route from food animal to humans through the food chain.

Our findings highlight the presence of *bla*_CTX-M-55_, *bla*_CTX-M-14_, *bla*_CTX-M-65_, *bla*_CTX-M-123_, *bla*_CTX-M-14b_, *bla*_CTX-M-15_, and *bla*_CTX-M-130_, which were the most common CTX-M type genes detected in patients with diarrhea [42]. This is the first study reporting the detection of the *bla*_CTX-M-130_ gene in *S*. Paratyphi B recovered from a 10-month-old boy's fecal sample. Overall, these results improved our understanding of the transmission dynamics of CTX-M genes, which are known to be carried on plasmids, between animals, humans and environments. Of the 775 beta-lactamase–producing isolates, 30.45% (*n* = 236) contained at least one QRDR. Our findings suggest that the incidence of PMQRs in *S. enterica* isolates is increasing (Fig. 3C and D), as previously reported [42]. Moreover, 12 QRDR point mutations, 6 in GyrA, 2 in GyrB, 3 in ParC, and 1 in ParE were found in 1537 isolates.

However, the emergence and rapid spread of mobile resistance genes mechanisms MCR-1 [49] and *tet*(X4) [50,51] which have gained global attention. In this study, a total of 27 *S. enterica* isolates were positive for *mcr* genes, accounting for 1.38% of all WGS isolates. However, the prevalence of *mcr-1* was 0.76% and 1.19% in 2010–2013 and 2014–2017, respectively. After the cessation of colistin use as a feed additive for animals in China from 30 April 2017, the prevalence of *mcr* in *Salmonella* may show a decreasing trend in the future. Similar to previous reports [52], *S*. Typhimurium and its variant was the most common serovar carrying *mcr* genes, accounting for 29.63% (8/27) and 33.33% (9/27), respectively. A recent genomic epidemiological study reported that serovars and STs were associated with a high rate of colistin resistance among *S. enterica* isolates [53]. Among these *mcr*-positive isolates, 10 STs including 1 novel ST were identified, with ST34 the predominant ST. We reported the identification of mobile tigecycline resistance gene *tet*(X4) in an *S. enterica* serovar Llandoff ST8300 isolate, which is a potential host for the spread of tigecycline resistance.

Recent studies [54,55] reported that a relatively high prevalence of *fosA7* (>9.00%) was identified in *Salmonella* isolates from food animals and retail meat products in China, which was consistent with our results. We also found that 2.9% of *Salmonella* isolates of human origin carried *fosA7*. To our knowledge, this is the first study that comprehensively describes the prevalence of *fosA7* in *Salmonella* from human and non-human origin sources in China.

Inappropriate use of antimicrobials in livestock production and human clinical medicine may contribute to the development and accumulation of AMR in pathogenic bacteria and is a major threat to public health. A recent study that compared AMR with antimicrobial usage data showed that the prevalence of resistance to fluoroquinolones and third-generation cephalosporins was correlated with usage [56]. Moreover, the broad patterns of antimicrobial use in the treatment of diarrhea across the Global Enteric Multicenter Study sites also showed that macrolides (azithromycin) and fluoroquinolones (in particular ciprofloxacin) were used more frequently in Asia [57].

Our work has several limitations. First, the dataset used in this study consisted of data collected for different reasons, during a varying time period and with different projects in mind. We cannot rule out sampling bias because the comprehensive network system is not built up yet, however, as far as we know, it is currently the largest database for *Salmonella* in China. We acknowledge the potential bias, which needs to be studied further. Among the strains isolated from patients and farm animals, we did not obtain a history of drug use. The previous study indicated higher rates of antibiotic resistance in high consuming countries [58]. Second, no data were available on total meat consumption including all meat types each year by provinces or municipal cities. Third, as for the 29 serovars mentioned above which have not been reported in China and their correlation with strains isolated from other countries were not explored. Fourth, no phenotypic data of antimicrobial susceptibility testing were available, AMR trends were explored based on WGS prediction. Another limitation is that we did not sequence all the 164 serovars of *S. enterica* strains in the Bacterium-learning Union, but selected 130 serovars of them. Moreover, *S*. 1,4, [5] : 12 : i : 1,2 and *S*. I 1,4, [5],12: i:— were not well distinguished in our database, we will refine this shortcoming in the next study. Last, we collected 35 382 *S. enterica* isolates from 23 provinces or municipal cities, but only provide WGS genomic data for 5.55% of the isolates. Moreover, WGS could not determine the precise location of ARGs, VFs, and MGEs, so long-read sequencing methods are needed in the future.

## CONCLUSION

In conclusion, our work provides the first systematic overview of current serovar prevalence and AMR trends of *S. enterica* strains isolated from human and non-human origins in China. *S*. Typhimurium, *S*. Enteritidis, and *S*. Derby were the main serovars responsible for human infection. Moreover, we have presented a most detailed genomic study of *S. enterica* in China during 2006–2019. Up till now, there is no comprehensive national surveillance scheme involving humans, food, animals, and the environment in China for continually monitoring AMR and geographical distribution of *S. enterica*. To our knowledge, our findings represent the largest longitudinal surveillance system for *S. enterica* in China and provides valuable public health knowledge on the trends and distribution of serovars and AMR in *S. enterica*. This large, observational study will provide valuable information for food safety interventions, control, and prevention of the development and spread of AMR *Salmonella enterica* in China.

## MATERIALS AND METHODS

### Dataset and study design

In this study, a total of 35 382 *S. enterica* isolates that had been obtained from various sources and geographical areas were used and analyzed. The detailed description of dataset and study design is summarized in Supplementary materials and methods. In order to explore the temporal and spatial dynamics of serovars and AMR, the correlation of strains between humans and food animals, the distinct features of serovars of gastro- and extra-intestinal infection of *S. enterica*, and evaluate the accuracy of traditional serology, we conducted a WGS study. Our final WGS data set comprised 1962 *S. enterica* isolates including human (*n* = 1159) and non-human origin (*n* = 803) from 22 provinces or municipal cities, ranging in date from 1982 to 2019 (Fig. 1, Fig. S5 and Table S2).

### WGS and gene content analysis

All 1972 *S. enterica* strains were subjected to WGS after cultivation for 18 h at 37°C and 180 rpm. Raw reads were processed with Trimmomatic version 0.36 [59], Unicycler version 0.4.7 [60], and Prokka version 1.13.3 [61], respectively, to generate high-quality reads, assembled contigs, and annotation of genome sequences. Individual accession numbers for assembled Illumina sequence data are available in Table S24.

The high-quality assembled contigs were screened against the SeqSero2 database, SISTR database, multilocus sequence typing database, ResFinder database, PointFinder database, Virulence Factor Database (VFDB), *Salmonella* Pathogenic Islands (SPIs) database, mobile genetic element database, and PlasmidFinder database using SeqSero2 version 1.1.1 [45], SISTR version 4.0.0 [46], MLST version 2.0 [29], ResFinder version 4.0 [62,63], ABRicate version 0.9.7 (https://github.com/tseemann/abricate), VFDB [64], SPIFinder version 2.0 [65], MobileElementFinder version 1.0.3 [66], and PlasmidFinder version 2.0.1 [67] to predict serovars, assign STs and detect acquired ARGs, plasmid replicons, genomic point mutations, virulence factors, SPIs, MGEs, and plasmid types, respectively, in each genome. PHASTER (http://phaster.ca/) [68] was used to predict phage-like regions in high-quality assembled contigs. The presence of *mcr-1, mcr-3, mcr-9*, and *tet*(X4) genes in *Salmonella* genomes was confirmed using polymerase chain reaction assays using primers from previous reports [49,51,69,70] and 2 × Phanta Master Mix (Code: P511-03, Vazyme Biotech) and Sanger sequencing.

### Mapping and phylogenetic analysis

Candidate SNPs were identified and filtered using BWA version 0.7.17 [71,72] and SAMtools version 1.10 [73]. GATK version 4.1.4 [74] and BCFtools [75] version 1.10 were used to convert SNPs into variant call format (VCF) and summarized into a VCF file. Maximum-likelihood (ML) phylogenetic trees were built from SNP alignments using FastTree version 2.1.1 [76]. The interactive tree of life (iTOL version 6.1.1, https://itol.embl.de/) [77] was used for phylogenetic tree visualization and annotation. The minimum spanning tree of multi-locus STs was generated in PHYLOViZ 2.0 (http://www.phyloviz.net/) [78].

### Statistical analyses and visualization

Statistical significance was taken at *P* < 0.05. Multiple comparisons were performed by the Kruskal–Wallis test and Mann–Whitney U test (unpaired *t*-test) using GraphPad Prism version 8.0. Venn diagrams were drawn in Venn Diagrams (http://bioinformatics.psb.ugent.be/webtools/Venn/). Easyfig (http://easyfig.sourceforge.net/) [79] was used to visualize the genetic context comparisons. The detailed description of materials and methods is summarized in Supplementary materials and methods.

## DATA AVAILABILITY

All assembled Illumina sequence data are available from the National Center for Biotechnology Information under the BioProject number: PRJNA766315. Sequence reads generated for this study have been submitted to the National Microbiology Data Center (NMDC) under BioProject number: NMDC10017893 and NMDC10018145. All data and database in this study are publicly available, the detailed description of data availability is summarized in Supplementary materials and methods.

## CODE AVAILABILITY

The source code used for *Salmonella* genome analysis is available in the following gitee repository: https://gitee.com/wangyanan999/salmonella_enterica.

## Supplementary Material

nwac269_Supplemental_Files

## Notes

Members are listed in the Acknowledgments section.
