# Supplementary material for: The temporal dynamics of antimicrobial-resistant Salmonella enterica and predominant serovars in China
Source: Natl Sci Rev. 2022 Nov 29;10(3):nwac269. doi: 10.1093/nsr/nwac269 (PMC10076184; doi:10.1093/nsr/nwac269)
Supplement: nwac269_Supplemental_Files [file nwac269_supplemental_files.zip › Supplementary_Figure_S1-30.pdf]

Supplementary figures for The temporal dynamics of antimicrobial-resistant-*Salmonella enterica* and predominant serovars in China

Yanan Wang[1,2,#], Yue Liu[3,#], Na Lyu[2], Zhiyuan Li[2], Sufang Ma[2], Demin Cao[2,4], Yuanlong Pan[2], Yongfei Hu[5], Hua Huang[6], George F. Gao[2,4,7,*], Xuebin Xu[3,*] on behalf of the Bacterium-learning Union[†], & Baoli Zhu[2,4,8,9,*]

[1]International Joint Research Center of National Animal Immunology, College of Veterinary Medicine, Henan Agricultural University, Zhengzhou, Henan 450046, China.

[2]CAS Key Laboratory of Pathogen Microbiology and Immunology, Institute of Microbiology, Chinese Academy of Sciences, Beijing 100101, China.

[3]Department of Microbiology, Shanghai Municipal Center for Disease Control and Prevention, Shanghai 200336, China.

[4]Savaid Medical School, University of Chinese Academy of Sciences, Beijing 100049, China.

[5]State Key Laboratory of Animal Nutrition, College of Animal Science and Technology, China Agricultural University, Beijing 100193, China.

[6]Beijing Products Quality Supervision and Inspection Institute, Beijing 101300, China.

[7]Chinese Center for Disease Control and Prevention (China CDC), Beijing 102206, China.

[8]Beijing Key Laboratory of Antimicrobial Resistance and Pathogen Genomics, Beijing 100101, China.

[9]Department of Pathogenic Biology, School of Basic Medical Sciences, Southwest Medical University, Luzhou, Sichuan 646000, China.

[#]These authors contributed equally to this work.

*These corresponding authors contributed equally to this work.

[†]Members are listed in the Acknowledgments section.

E-mail address: gaof@im.ac.cn (G. Gao), xuxuebin@scdc.sh.cn (X. Xu), or zhubaoli@im.ac.cn (B. Zhu)

Supplementary figures

Fig. S1. The proportions of Typhoid, paratyphoid, and non-typhoidal *Salmonella*.

Fig. S2. Geographical distribution and characteristics of *S. enterica* serovars in China.

Fig. S3. Changes in *S*. Typhi isolates between 2006 and 2019.

Fig. S4. A, Number of serovars in *S. enterica* isolates between 2006 and 2019. B, Number of new serovars of *S. enterica* isolates identified over the previous year.

Fig. S5. Summary of 1,962 WGS *S. enterica* isolates.

Fig. S6. Dominant serovar switch in WGS *Salmonella* isolates during 2006–2017.

Fig. S7. The epidemic of *S*. 1,4,[5]:12:i:1,2 and *S*. I 1,4,[5],12:i:- strains of human origin in China.

Fig. S8. Minimum spanning tree of the 1,962 *Salmonella* genomes by multi-locus sequence typing.

Fig. S9. The prevalence and diversity of STs in WGS *S. enterica* genomes.

Fig. S10. Temporal changes of MDR-*Salmonella* rates in pig and chicken in different time groups.

Fig. S11. Comparison of the genetic environment of *mcr* genes.

Fig. S12. The correlation of AMR genes, VFs and MGEs.

Fig. S13. The prevalence of plasmid replicons in NTS isolates.

Fig. S14. Shared and distinct features of *S. enterica* serovars of human and non-human origin.

Fig. S15. A-C, The distribution of numbers of (A) ARGs, (B) VFs, and (C) MGEs in each NTS isolate between human and non-human origin.

Fig. S16. The proportion of AMR-*Salmonella* isolates between human and non-human origin.

Fig. S17. The proportion of MDR-*Salmonella* isolates from non-human origin.

Fig. S18. Shared and distinct features of *S. enterica* strains isolated from humans, wet markets, and the environment in Shanghai.

Fig. S19. Characteristics of *S. enterica* isolates of bloodstream and diarrhea infection

in China.

Fig. S20. A-B, Comparison of (A) ARGs and (B) VFs between Gastro- and Extra-intestinal infection *S.* Enteritidis.

Fig. S21. Summary of *S*. Enteritidis strains of human origin in China.

Fig. S22. Geographical origin of 550 *S*. Kentucky isolates in China.

Fig. S23. Geographical origin of eight S. Chester isolates in China.

Fig. S24. Phylogenetic analysis of 338 *S*. Typhimurium genomes.

Fig. S25. Phylogenetic analysis of 164 *S*. I 1,4,[5],12:i:- genomes.

Fig. S26. Phylogenetic analysis of *S*. Enteritidis, based on 6,976 SNPs.

Fig. S27. Molecular phylogenetic analysis of *S*. Choleraesuis isolates, based on SNP differences.

Fig. S28. Phylogenetic analysis of *S*. Heidelberg, based on 3,082 SNPs.

Fig. S29. Phylogenetic analysis of *S*. Typhi, based on 8,772 SNPs

Fig. S30. Phylogenetic analysis of *S*. *enterica* serovar Paratyphi B and its variants.

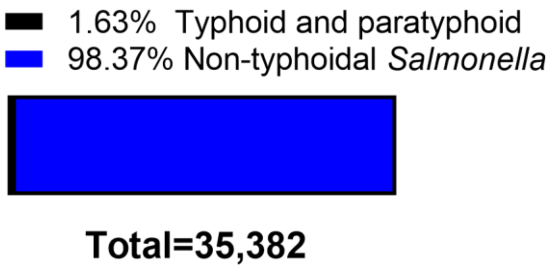

Fig. S1. The proportions of Typhoid, paratyphoid, and non-typhoidal *Salmonella*.

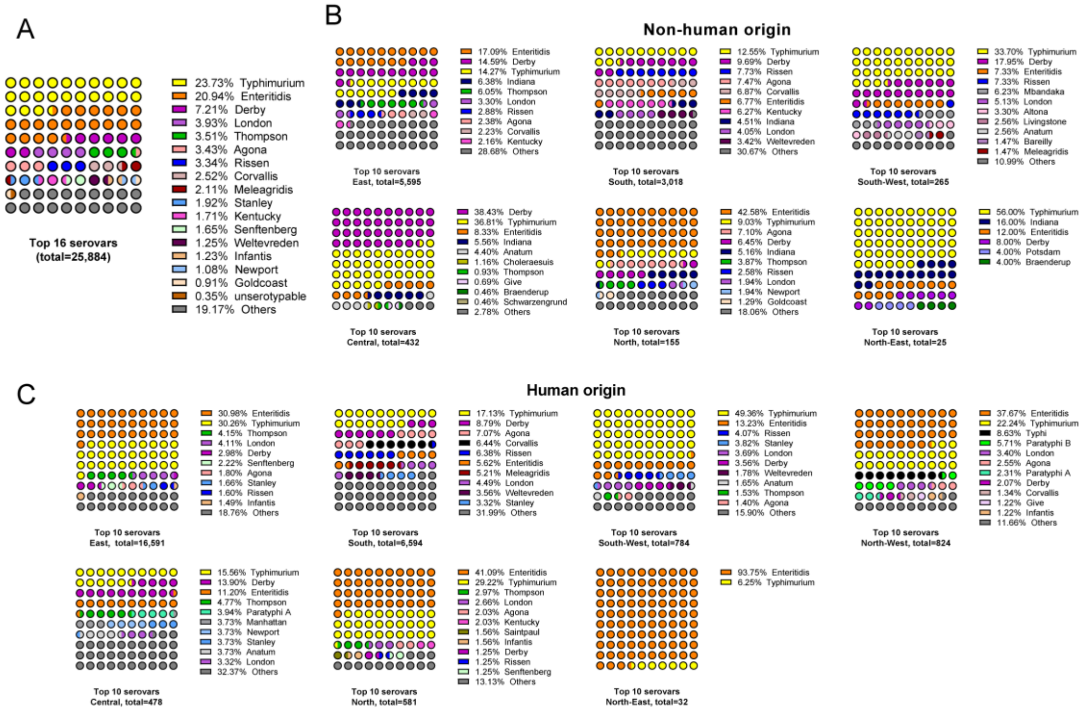

Fig. S2. Geographical distribution and characteristics of *S. enterica* serovars in China.

A, Top 16 serotypes of *S. enterica* in China. B, Serovar proportions of *S. enterica* isolates of human origin in different regions in China. C, Serovar proportions of *S. enterica* isolates of non-human origin in different regions in China. The regions included East, South, South-West, Central, North, North-West, and North-East.

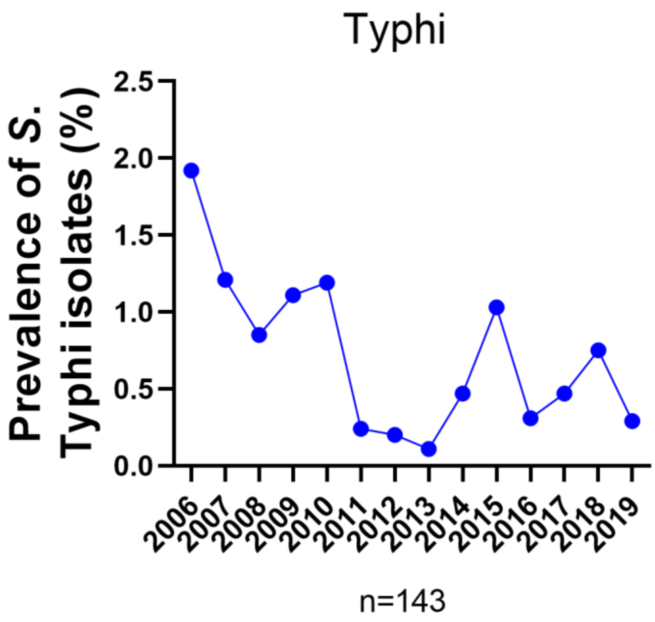

Fig. S3. Changes in *S*. Typhi isolates between 2006 and 2019.

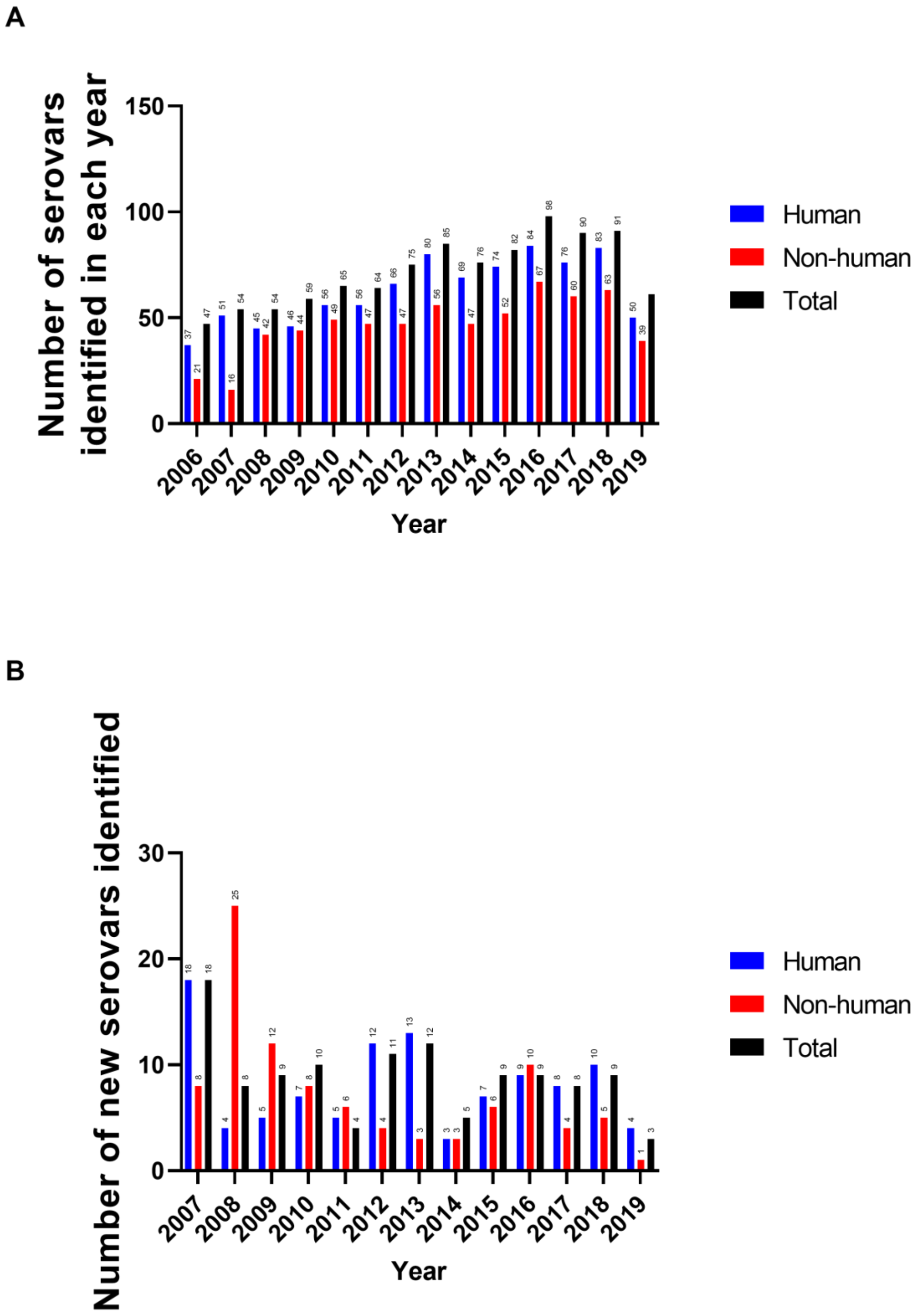

Fig. S4. A, Number of serovars in *S. enterica* isolates between 2006 and 2019. B, Number of new serovars of *S. enterica* isolates identified over the previous year.

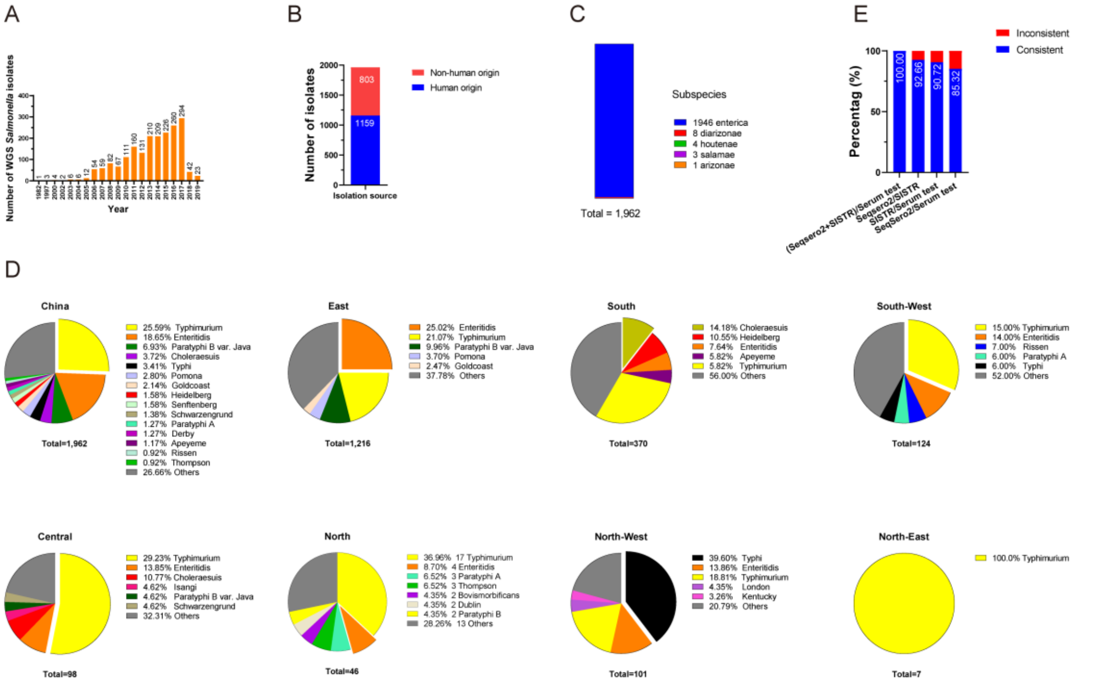

Fig. S5. Summary of 1,962 WGS *S. enterica* isolates.

A, Distribution of the 1,962 WGS *S. enterica* isolates. B, Isolation source of 1,962 *S. enterica* isolates. Of these isolates, 59.07% (1,159/1,962) and 40.93% (803/1,962) were the human origin and non-human origin, respectively. C, The 1,962 *S. enterica* isolates were divided into five subspecies. D. Serovar proportions of 1,962 WGS *S. enterica* isolates in different regions in China. The regions included East, South, South-West, Central, North, North-West, and North-East. E, Comparison of conventional serotyping, SeqSero2, and SISTR prediction results of *S. enterica* isolates.

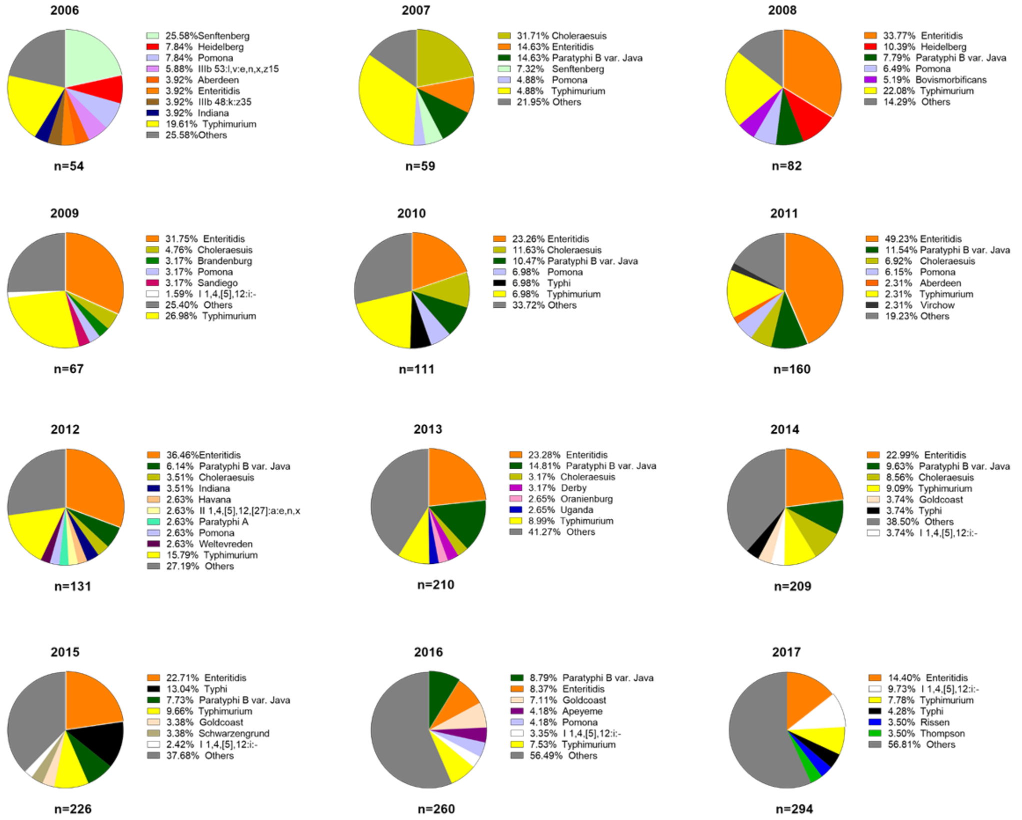

Fig. S6. Dominant serovar switch in WGS *Salmonella* isolates during 2006–2017.

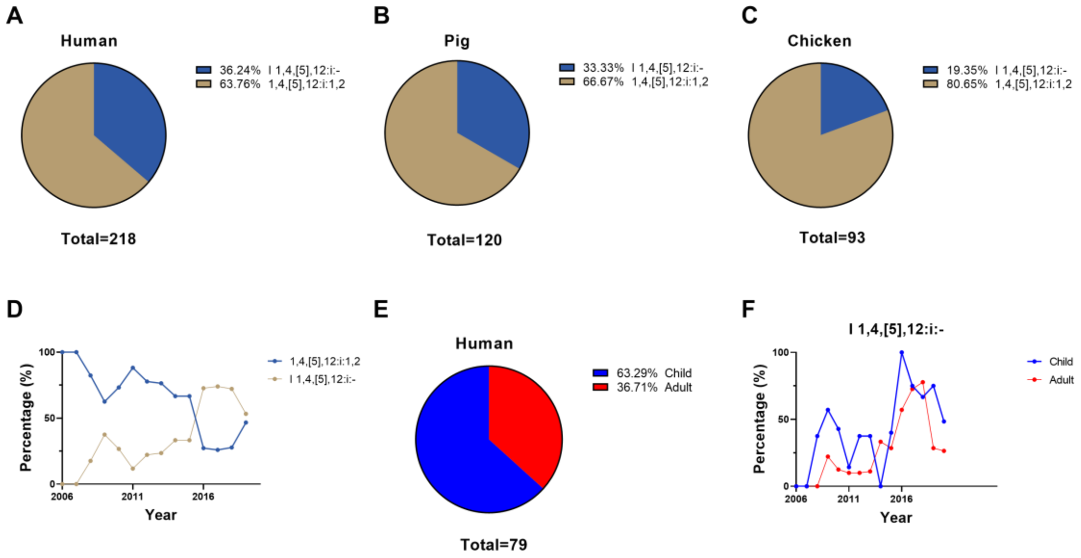

Fig. S7. The epidemic of *S*. 1,4,[5]:12:i:1,2 and *S*. I 1,4,[5],12:i:- strains of human origin in China.

A-C, Comparison of the prevalence of *S*. 1,4,[5]:12:i:1,2 (biphasic *S*. Typhimurium) and its monophasic variant I 1,4,[5],12:i:- strains in (A) human, (B) pig, and (C) chicken. D, Temporal changes of *S*. 1,4,[5]:12:i:1,2 and its monophasic variant I 1,4,[5],12:i:- strains in humans. E, Comparison of the prevalence of *S*. I 1,4,[5],12:i:- strains between child and adult. F, Temporal changes of *S*. I 1,4,[5],12:i:- strains in child and adult. Each point represents a proportion rate of *S*. 1,4,[5]:12:i:1,2 and *S*. I 1,4,[5],12:i:- in each year. The isolation source of isolates is indicated by different colors.

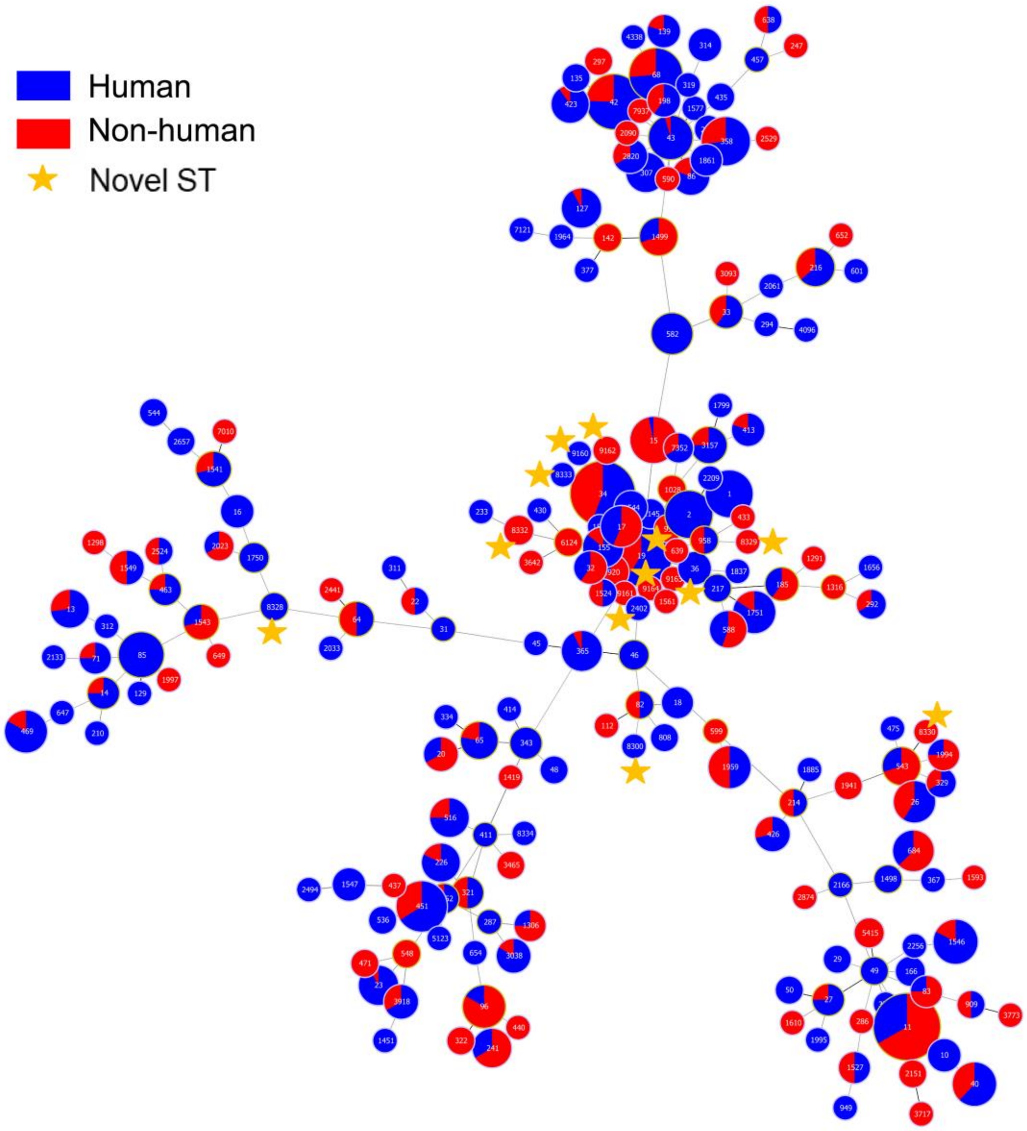

Fig. S8. Minimum spanning tree of the 1,962 *Salmonella* genomes by multi-locus sequence typing.

The isolation source of all isolates is indicated by different colors. Each node represents a distinct ST. Node size is proportional to the number of strains represented. Human: blue color. Non-human: red color. Novel ST: yellow pentacle.

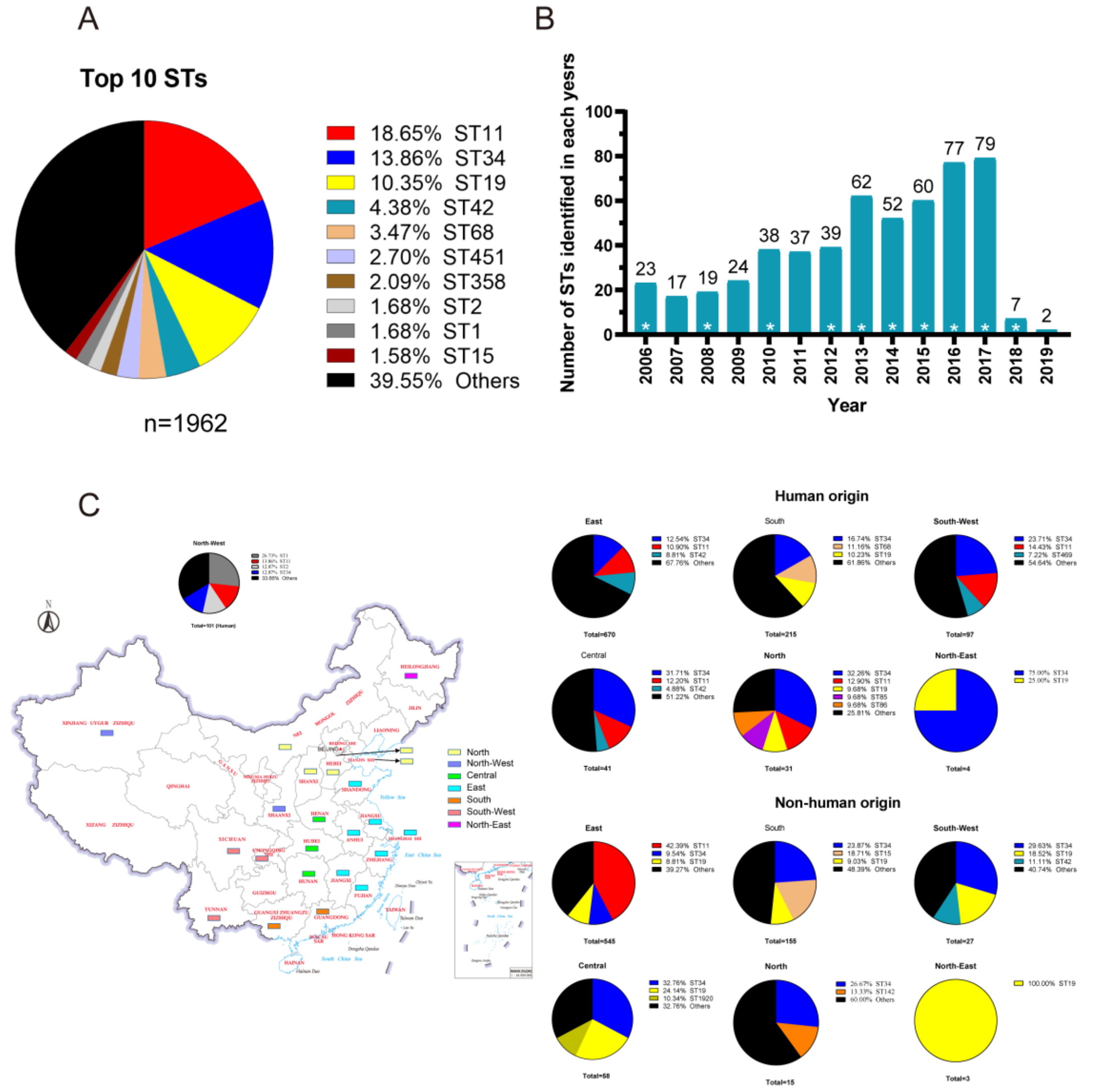

Fig. S9. The prevalence and diversity of STs in WGS *S. enterica* genomes.

A, Top 10 STs among the 1,962 WGS *S. enterica* isolates. B, Diversity of STs of *S. enterica* isolates between 2006 and 2019. The “*” means, new ST was identified. C, Distribution and characteristics of dominant STs in sampling regions. The regions included East, South, South-West, Central, North, North-West, and North-East.

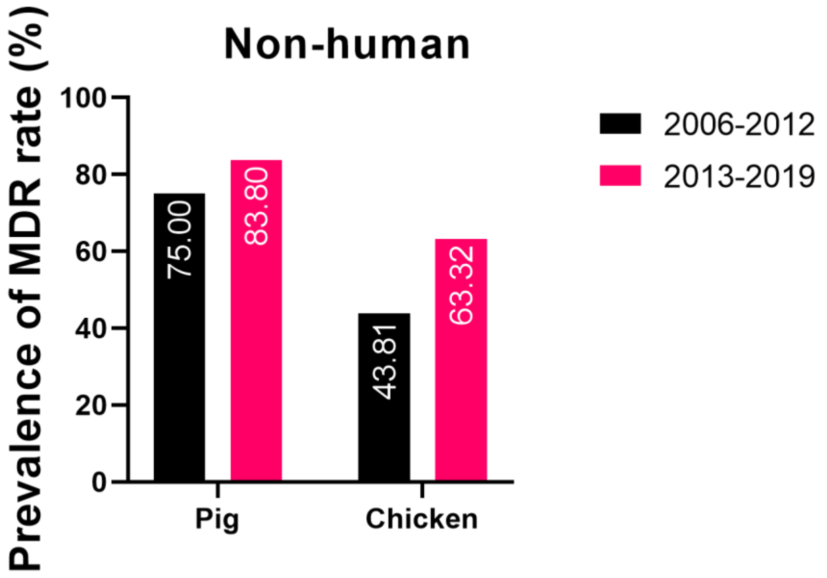

Fig. S10. Temporal changes of MDR-*Salmonella* rates in pig and chicken in different time groups.

The number of MDR-*Salmonella* strains in chickens and pigs is 230 and 146, respectively.

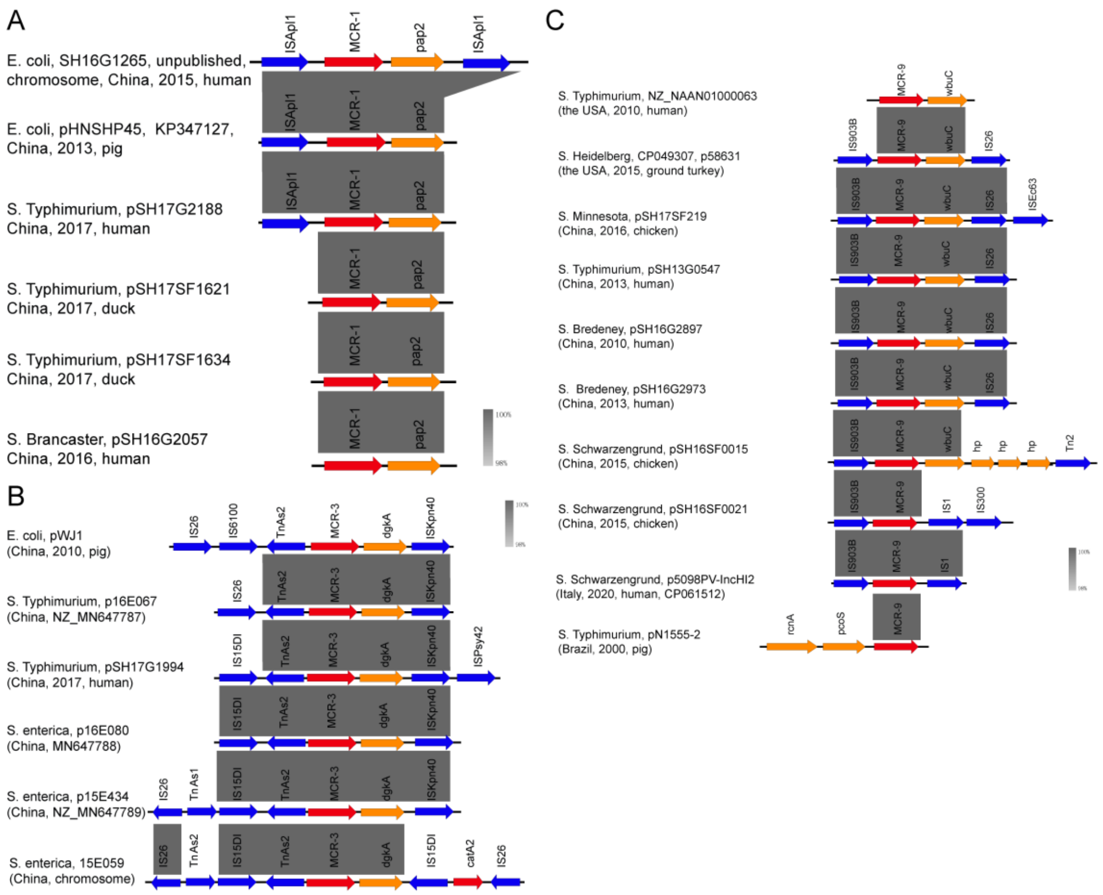

Fig. S11. Comparison of the genetic environments of *mcr* genes.

A, Comparison of the genetic environments of the *mcr-1* gene. B, Comparison of the genetic environments of the *mcr-3* gene. C, Comparison of the genetic environments of the *mcr-9* gene. Colored arrows indicate open reading frames, with yellow, dark blue, and red arrows representing others, mobile elements, and ARGs, respectively. Regions of >98% identity are indicated by dark grey shading.

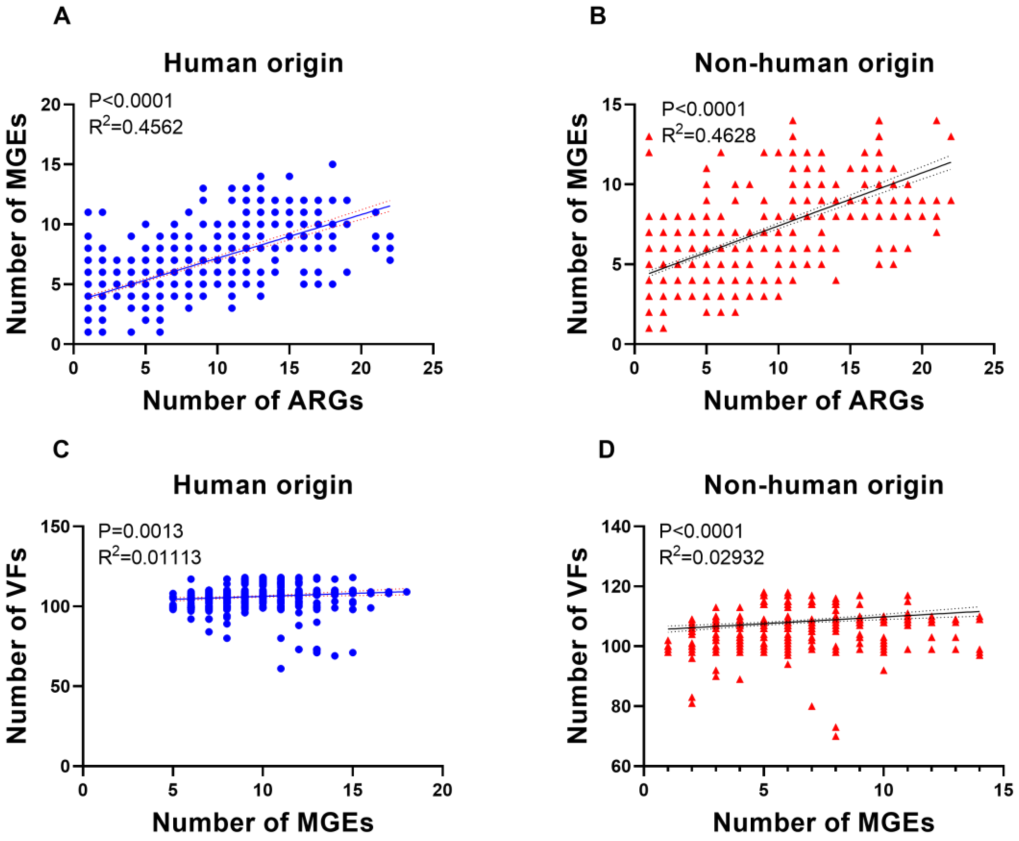

Fig. S12. The correlation of AMR genes, VFs and MGEs.

A, The correlation of ARGs and MGEs detected from genomes of human origin (n=930). B, The correlation of ARGs and MGEs detected from genomes of non-human origin (n=775). C, The correlation of VFs and MGEs detected from genomes of human origin (n=930). D, The correlation of VFs and MGEs detected from genomes of non-human origin (n=773). Solid black lines represent the fitted prevalence from liner fitting model analysis and shading the 95% confidence intervals.

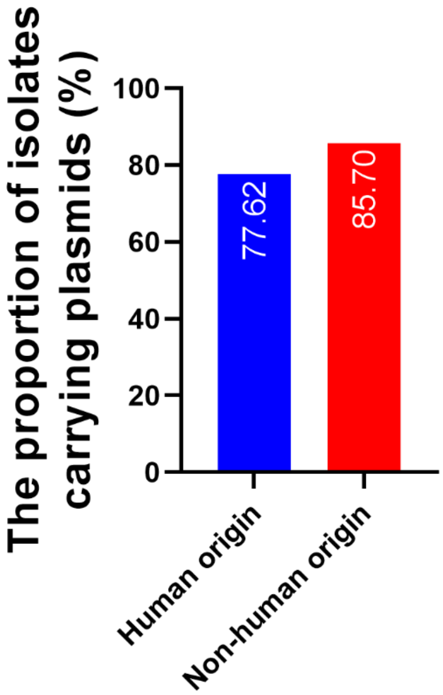

Fig. S13. The prevalence of plasmid replicons in NTS isolates.

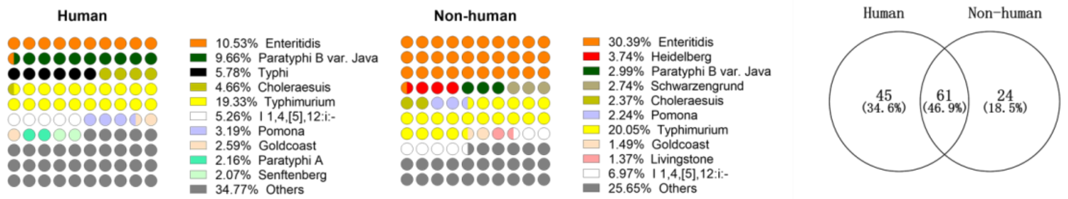

Fig. S14. Shared and distinct features of *S. enterica* serovars of human and non-human origin.

A, Dominant serovars of *S. enterica* strains isolated from humans. B, Dominant serovars of *S. enterica* isolates from non-human. C, Shared and distinct serovars between human and non-human origin. A total of 61 serovars were shared between the two groups

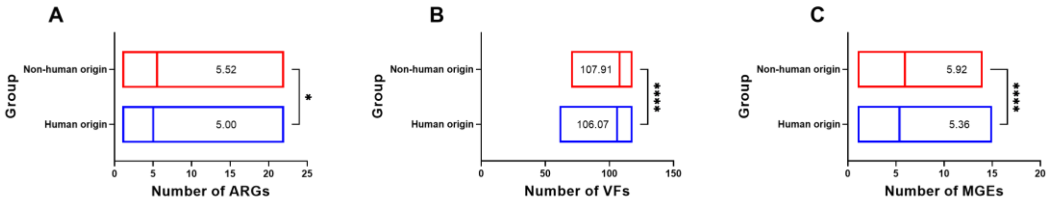

Fig. S15. A-C, The distribution of numbers of (A) ARGs, (B) VFs, and (C) MGEs in each NTS isolate between human and non-human origin.

Mann Whitney U test (unpaired t-test) was performed using GraphPad Prism version 8.0. The ‘*’ on the right represent *P*-values. *: $P < 0.05$; **: $P < 0.01$; ***: $P < 0.001$.

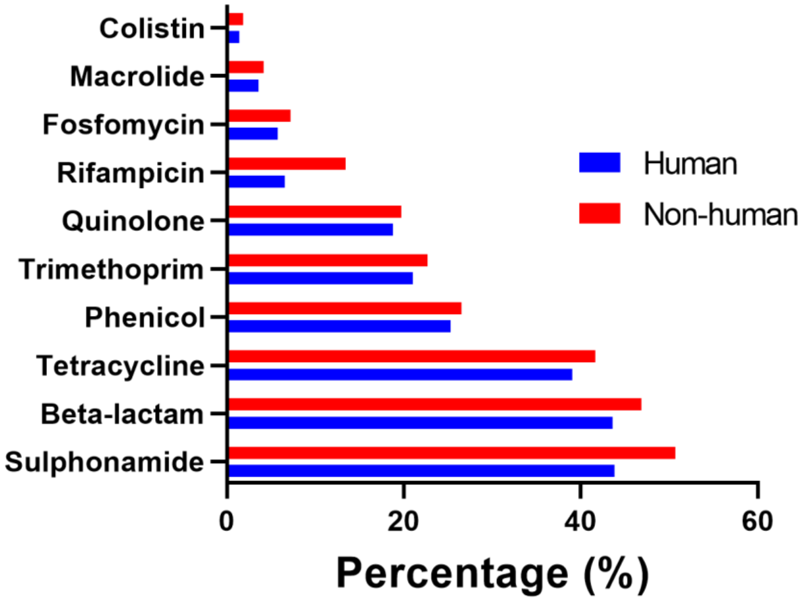

Fig. S16. The proportion of AMR-*Salmonella* isolates between human and non-human origin.

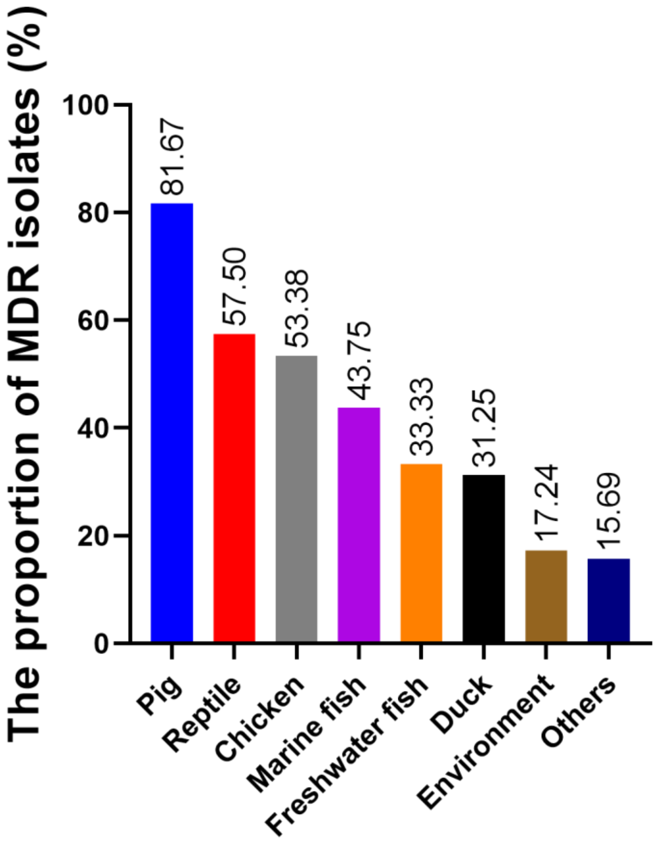

Fig. S17. The proportion of MDR-*Salmonella* isolates from non-human origin.

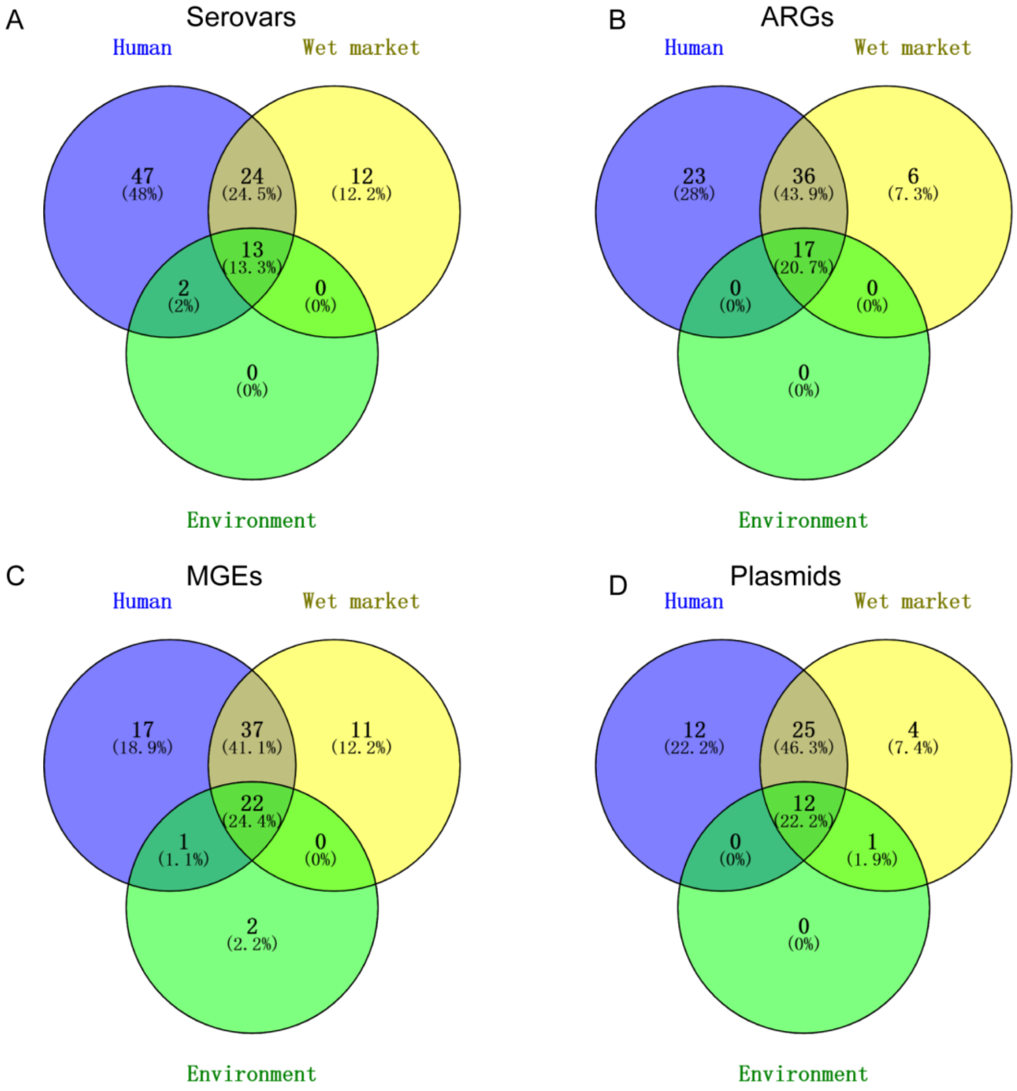

Fig. S18. Shared and distinct features of *S. enterica* strains isolated from humans, wet markets, and the environment in Shanghai.

A, Serovars. B, ARGs. C, MGEs. D, Plasmid replicons.

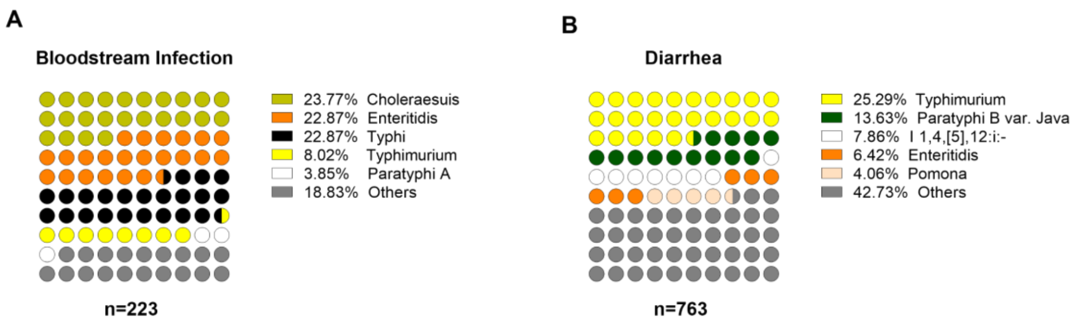

Fig. S19. Characteristics of *S. enterica* isolates of bloodstream and diarrhea infection in China.

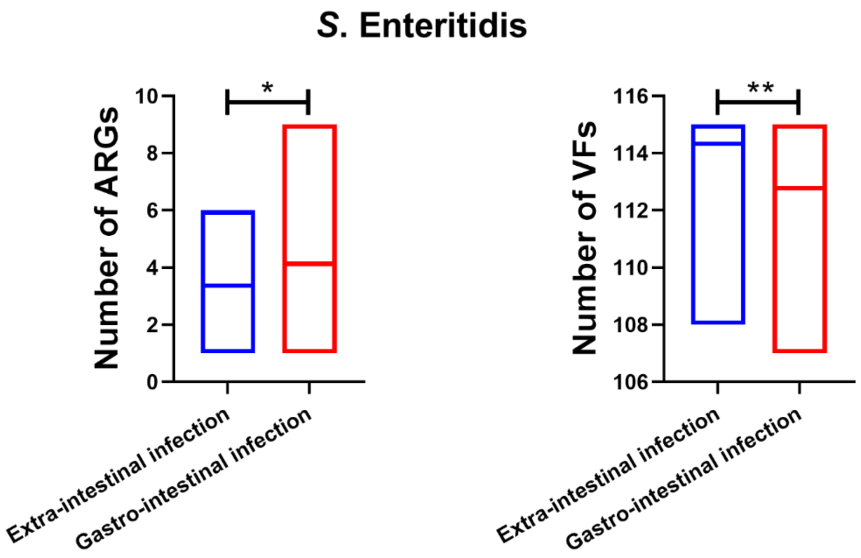

Fig. S20. A-B, Comparison of (A) ARGs and (B) VFs between Gastro- and Extra-intestinal infection *S.* Enteritidis.

Mann Whitney U test (unpaired t-test) was performed using GraphPad Prism version 8.0. The '*' on the top represent *P*-values. *: $P < 0.05$; **: $P < 0.01$; ***: $P < 0.001$.

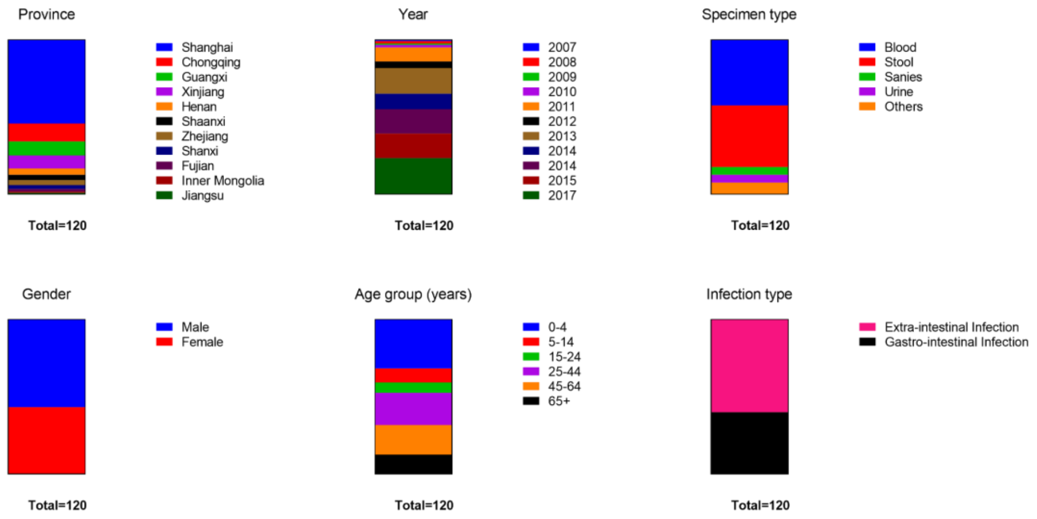

Fig. S21. Summary of *S*. Enteritidis strains of human origin in China.

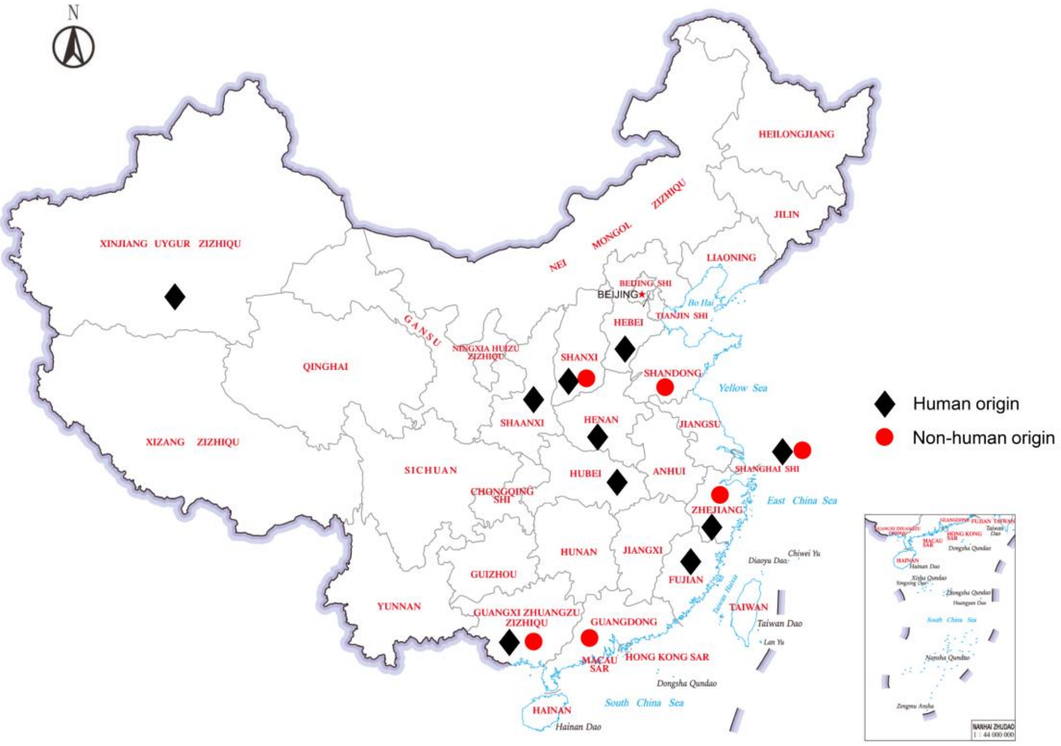

Fig. S22. Geographical origin of 550 *S*. Kentucky isolates in China.

294 (1.14 %) of human origin strains isolated from ten provinces or municipal cities (including Guangxi, Fujian, Zhejiang, Hebei, Henan, Hubei, Shanxi, Shaanxi, Shanghai, and Xinjiang), and 256 (2.70%) of non-human origin strains from six provinces (including Guangxi, Guangdong, Shanghai, Zhejiang, Shanxi, and Shandong).

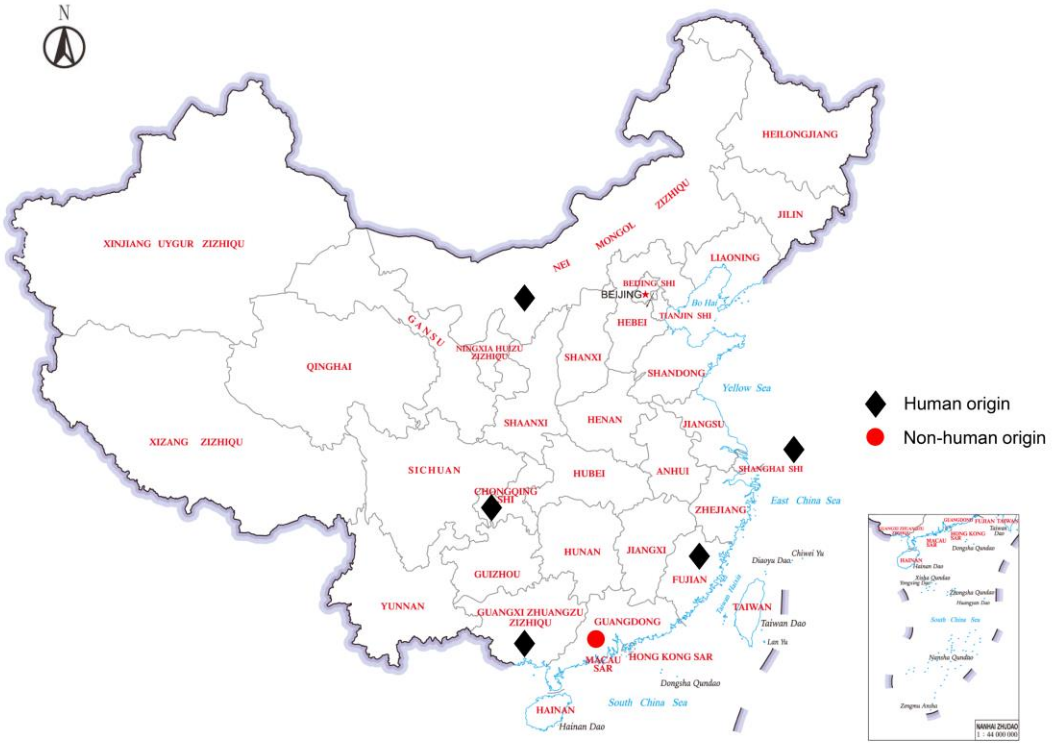

Fig. S23. Geographical origin of eight *S*. Chester isolates in China.

A total of eight *S*. Chester strains were identified, including seven and one strain isolated from human origin and non-human origin (freshwater fish), respectively.

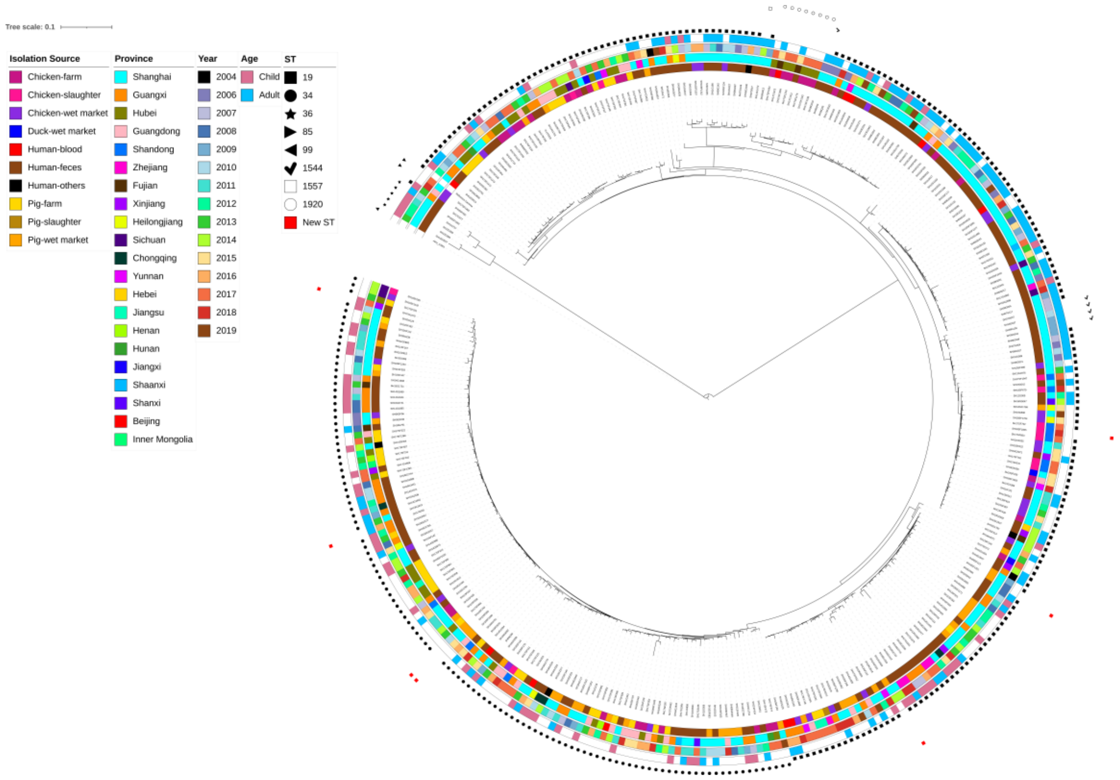

Fig. S24. Phylogenetic analysis of 338 *S*. Typhimurium genomes.

Maximum-likelihood phylogenetic tree based on the 338 genome sequences from this study. Sequencing reads were mapped to the complete genome sequence of *S*. Typhimurium str. LT2. *S*. Paratyphi A strain AKU1_12601 was used as an outgroup. The tree is based on 43,669 chromosomal SNPs. From the inner to outer circles are 1. Isolation source, 2. Province, 3. Year, 4. Age group, 5. ST of the isolates.

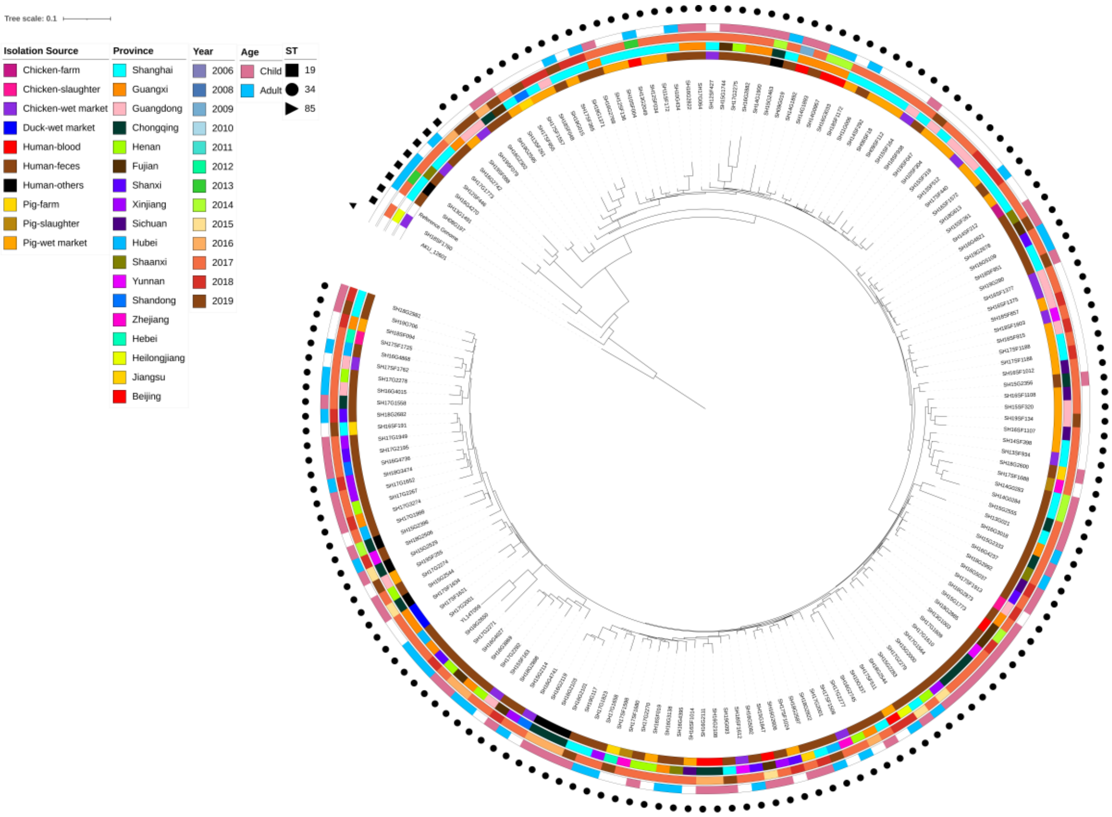

Fig. S25. Phylogenetic analysis of 164 *S*. I 1,4,[5],12:i:- genomes.

Maximum-likelihood phylogenetic tree based on the 164 genome sequences from this study. Sequencing reads were mapped to the complete genome sequence of *S*. Typhimurium str. LT2. *S*. Paratyphi A strain AKU1_12601 was used as an outgroup. The tree is based on 14,291 chromosomal SNPs. From the inner to outer circles are 1. Isolation source, 2. Province, 3. Year, 4. Age group, 5. ST of the isolates.

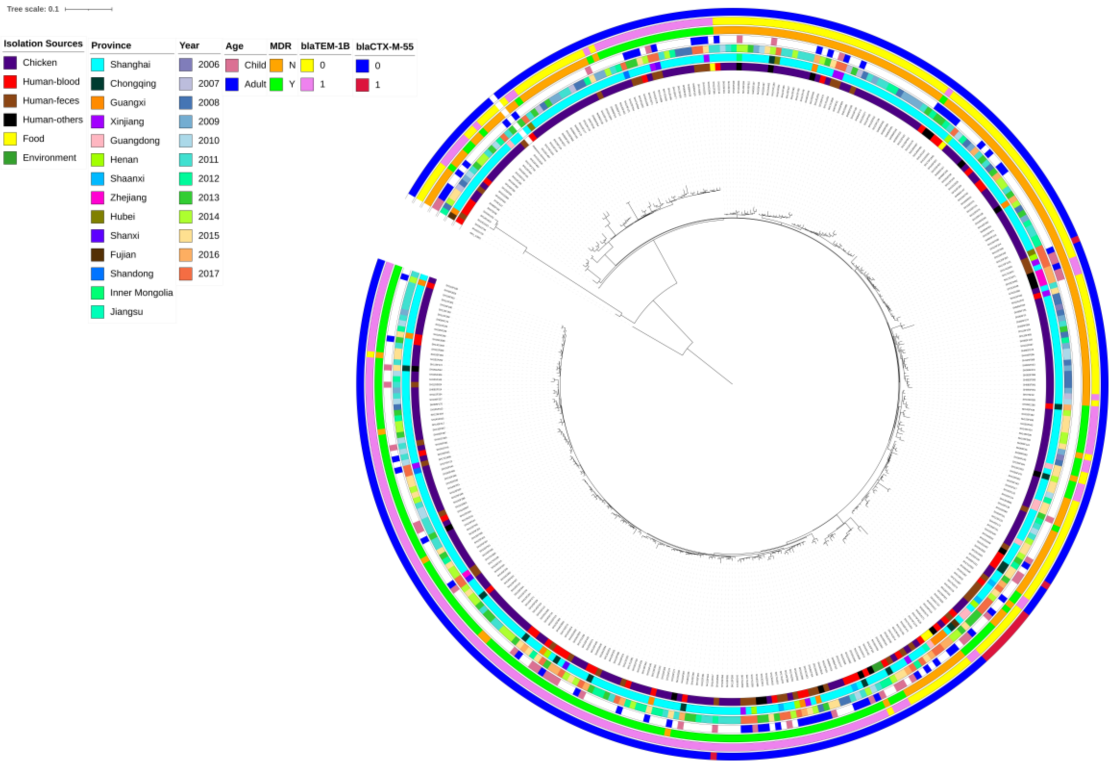

Fig. S26. Phylogenetic analysis of *S*. Enteritidis, based on 6,976 SNPs.

Maximum-likelihood phylogenetic tree based on the 364 genome sequences from this study. Sequencing reads were mapped to the complete genome sequence of *S*. Enteritidis str. P125109. *S*. Paratyphi A strain AKU1_12601 was used as an outgroup. The tree is based on 6,976 chromosomal SNPs. From the inner to outer circles are 1. Isolation source, 2. Province, 3. Year, 4. Age group, 5-7. Presence of multidrug resistance (MDR) genotype, *bla*$_{TEM-1B}$, *bla*$_{CTX-M-55}$. N: no. Y, yes. “1” means presence. “0” means absence.

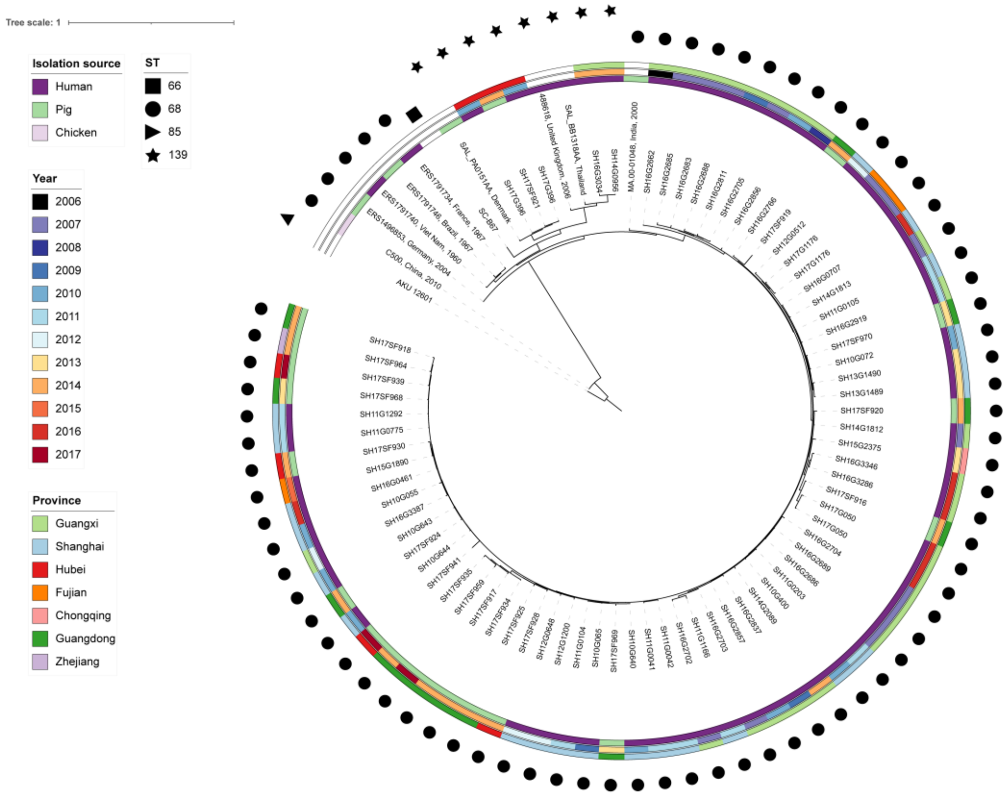

Fig. S27. Molecular phylogenetic analysis of *S*. Choleraesuis isolates, based on SNP differences.

Maximum-likelihood phylogenetic tree based on the 73 genome sequences from this study and nine publicly available *S*. Choleraesuis isolates. Sequencing reads were mapped to the complete genome sequence of *S*. Choleraesuis strain SC-B67. *S*. Paratyphi A strain AKU1_12601 was used as an outgroup. The tree is based on 4,905 chromosomal SNPs. From the inner to outer circles are 1. Isolation source, 2. Year, 3. Province, 4. ST of the isolates.

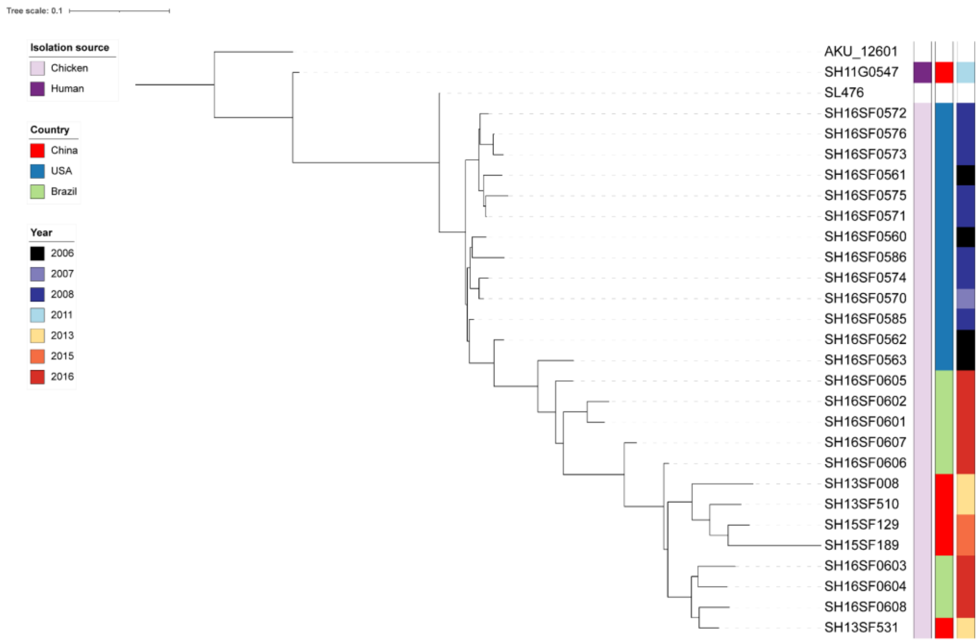

Fig. S28. Phylogenetic analysis of *S*. Heidelberg, based on 3,082 SNPs.

Maximum-likelihood phylogenetic tree based on the 27 genome sequences from this study. Sequencing reads were mapped to the complete genome sequence of *S.* Heidelberg str. SL476. *S.* Paratyphi A strain AKU1_12601 was used as an outgroup. The tree is based on 3,082 chromosomal SNPs. From the inner to outer circles are 1. Isolation source, 2. Country, 3. Year of the isolates.

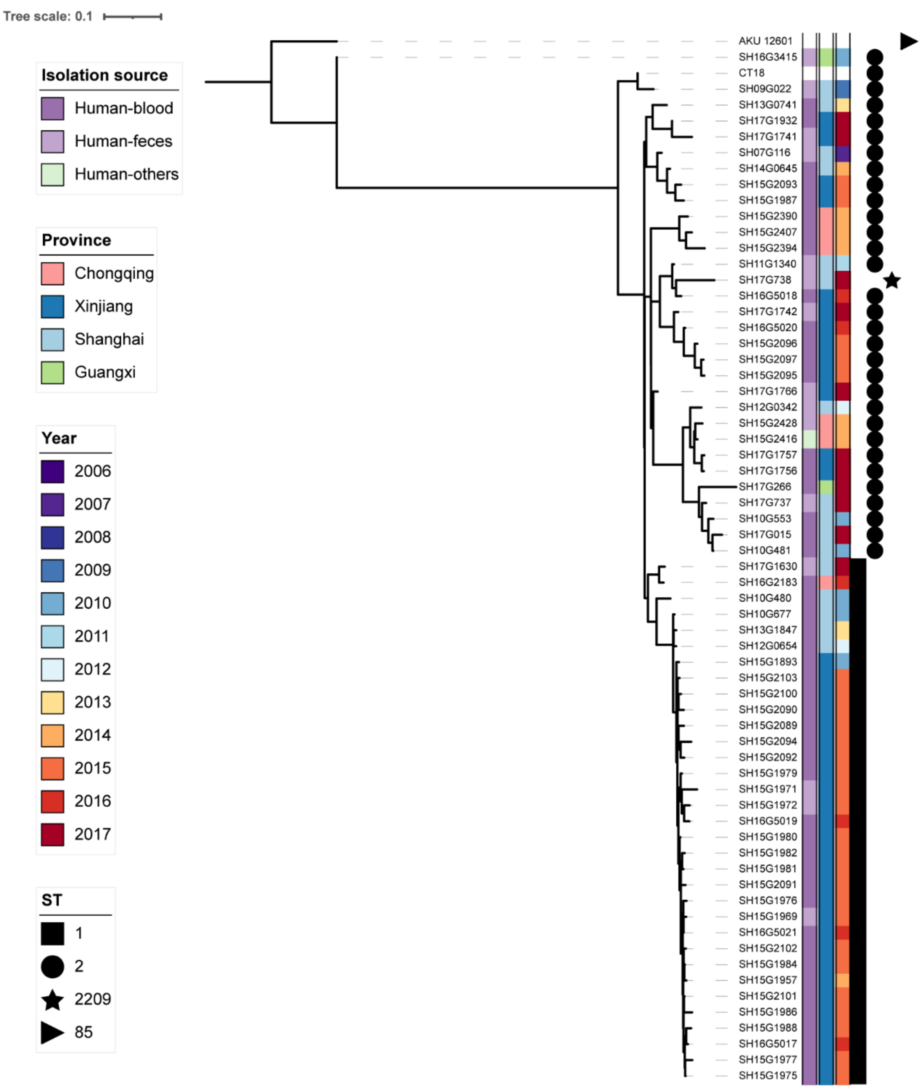

Fig. S29. Phylogenetic analysis of *S*. Typhi in China, 2006-2017, based on 8,772 SNPs.

Maximum-likelihood phylogenetic tree based on the 64 genome sequences from this study. Sequencing reads were mapped to the complete genome sequence of *S*. Typhi str. CT18. *S*. Paratyphi A strain AKU1_12601 was used as an outgroup. The tree is based on 8,772 chromosomal SNPs. From the inner to outer circles are 1. Isolation source, 2. Year, 3. Province, 4. ST of the isolates.

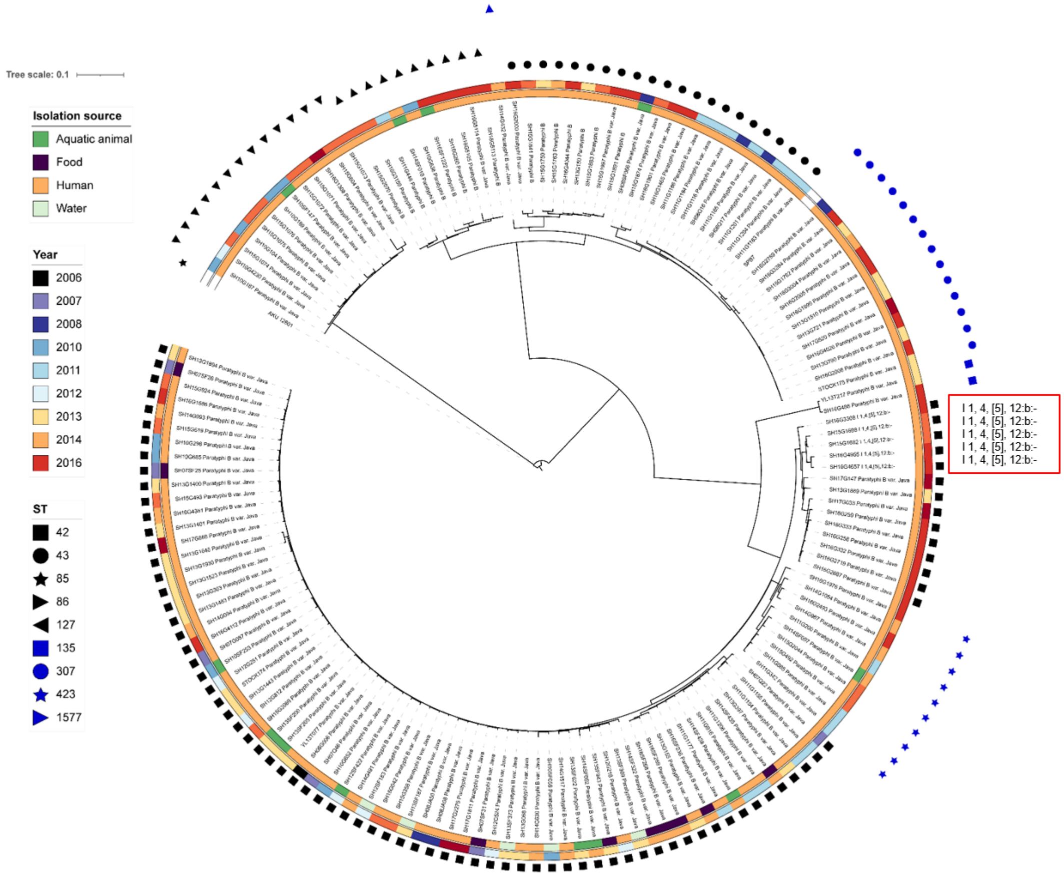

Fig. S30. Phylogenetic analysis of *S. enterica* serovar Paratyphi B and its variants, based on 87,271 SNPs.

Maximum-likelihood phylogenetic tree based on the 155 genome sequences from this study. Sequencing reads were mapped to the complete genome sequence of *S.* Paratyphi B str. SPB7. *S.* Paratyphi A strain AKU1_12601 was used as an outgroup. The tree is based on 87,271 chromosomal SNPs. From the inner to outer circles are 1. Isolation source, 2. Year, 3. ST of the isolates.
