# Supplementary material for: The temporal dynamics of antimicrobial-resistant Salmonella enterica and predominant serovars in China
Source: Natl Sci Rev. 2022 Nov 29;10(3):nwac269. doi: 10.1093/nsr/nwac269 (PMC10076184; doi:10.1093/nsr/nwac269)
Supplement: nwac269_Supplemental_Files [file nwac269_supplemental_files.zip › Supplementary_Materials_and_methods.docx]

Supplementary Materials and methods for The temporal dynamics of antimicrobial-resistant-*Salmonella enterica* and predominant serovars in China

Yanan Wang^1,2,#^, Yue Liu^3,#^, Na Lyu^2^, Zhiyuan Li^2^, Sufang Ma^2^, Demin Cao^2,4^, Yuanlong Pan^2^, Yongfei Hu^5^, Hua Huang^6^, George F. Gao^2,4,7,*^, Xuebin Xu^3,*^ on behalf of the Bacterium-learning Union^†^, & Baoli Zhu^2,4,8,9,*^

^1^International Joint Research Center of National Animal Immunology, College of Veterinary Medicine, Henan Agricultural University, Zhengzhou, Henan 450046, China.

^2^CAS Key Laboratory of Pathogen Microbiology and Immunology, Institute of Microbiology, Chinese Academy of Sciences, Beijing 100101, China.

^3^Department of Microbiology, Shanghai Municipal Center for Disease Control and Prevention, Shanghai 200336, China.

^4^Savaid Medical School, University of Chinese Academy of Sciences, Beijing 100049, China.

^5^State Key Laboratory of Animal Nutrition, College of Animal Science and Technology, China Agricultural University, Beijing 100193, China.

^6^Beijing Products Quality Supervision and Inspection Institute, Beijing 101300, China.

^7^Chinese Center for Disease Control and Prevention (China CDC), Beijing 102206, China.

^8^Beijing Key Laboratory of Antimicrobial Resistance and Pathogen Genomics, Beijing 100101, China.

^9^Department of Pathogenic Biology, School of Basic Medical Sciences, Southwest Medical University, Luzhou, Sichuan 646000, China.

^#^These authors contributed equally to this work.

*These corresponding authors contributed equally to this work.

^†^Members are listed in the Acknowledgments section.

E-mail address: [gaof@im.ac.cn](mailto:gaof@im.ac.cn) (G. Gao), xuxuebin@scdc.sh.cn (X. Xu), or [zhubaoli@im.ac.cn](mailto:zhubaoli@im.ac.cn) (B. Zhu)

# Materials and methods

## Dataset and study design

In this study, the data on the distribution of *Salmonella* serovars in China were obtained from the Chinese Local Surveillance System for *Salmonella* database (Bacterium-learning Union) from the period of September 15, 1982 and September 28, 2019. A total of 35,382 *S*. *enterica* isolates (Fig. 1A) that had been obtained from various sources and geographical areas were used and analyzed. The isolates originated from the collections of the Bacterium-learning Union including human (n=25,884) and non-human origins (n=9,498). The Bacterium-learning Union includes the *Salmonella* isolate database for the Center for Disease Control and Prevention (CDC) in over 20 provinces or municipal cities in China since 1 January 2006 led by Shanghai Municipal CDC. As of February 14, 2020, the Bacterium-learning Union has recorded over 50,000 *S*. *enterica* isolates from human, animal, and environmental samples over recent decades^1^. Human-associated isolates originated from blood, urine, stool (including inpatients and healthy carriers), and others, all of them were from public health laboratories in CDC and sentinel clinical laboratories from general hospitals that participated in the Bacterium-learning Union. Non-human isolates originated from the wet market, farm, slaughter food, and environmental sources, all of them were from public health laboratories in CDC and sentinel laboratories from universities. Wet market samples included swabs from live animals (such as fish, pets, aquatic and wild animals), animal products (for example, chicken, duck, goose, pork, beef, eggs), feed, vegetables, and others. Epidemiological and basic clinical data were recorded for each *Salmonella* isolate, including source, area, province, date, age, sex, etc.

In order to explore the temporal and spatial dynamics of serovars and AMR, the correlation of strains between humans and food animals, the distinct features of serovars of Gastro- and Extra-intestinal infection of *S*. *enterica*, and evaluate the accuracy of traditional serology (including serotyped, serovar variants, non-serotyped, and serovar has not been reported in China), we conducted a WGS study. Then, random subsampling was performed using a random number generator (Excel, Microsoft) and selected samples were collected and resuscitated from the stab cultures at Shanghai CDC. Our final WGS data set comprised 1,962 *S*. *enterica* isolates including human (n=1,159) and non-human origin (n=803) from 22 provinces or municipal cities, ranging in date from 1982 to 2019 (Fig. 1, Fig. S5 and Table S2). All isolates from the Bacterium-learning Union were maintained as stab cultures at room temperature and have not been manipulated since being placed in storage (generally 1-2 weeks after isolation from samples). All other isolates were stored as freeze-dried cultures.

## *Salmonella*-infection case definition

We defined a *Salmonella*-infection case as a person living in China with laboratory-confirmed infection with *Salmonella* reported between September 1, 1991, and September 28, 2019.

## Serotyping

Serological assays were performed according to the Kauffmann-White Scheme with O- and H-antigen specific sera by slide agglutination with commercial antiserum for each *Salmonella* isolate (Statens Serum Institute, Denmark), and a total of 164 serovars were identified.

## DNA extraction and WGS

All 1,972 *S*. *enterica* strains were subjected to WGS after cultivation for 18 h at 37 ℃ and 180 rpm. Afterward, total genomic DNA was extracted from each isolate using the Wizard Genomic DNA Extraction Kit (Promega, Beijing, China), and quantified using a Qubit Fluorometer 3.0. Illumina Nextera XT DNA Libraries were prepared according to the manufacturers’ instructions and sequenced using Illumina HiSeq 2500 platform to generate 150 bp paired-end reads. Raw FASTQs were subjected to quality control before downstream analysis. Raw reads were processed with Trimmomatic version 0.36 (https://github.com/usadellab/Trimmomatic)^2^, adapters and low-quality regions were removed, and reports before and after read trimming were generated. Ten genomes that failed quality control were excluded. Assembly and annotation of genome sequences were performed using Unicycler version 0.4.7 (<https://github.com/rrwick/Unicycler>)^3^ and Prokka version 1.13.3 (https://github.com/tseemann/prokka)^4^, respectively. After genome assembly, the number of contigs, and protein-coding sequence (CDS) of each isolate are summarized in Table S2. Individual accession numbers for assembled Illumina sequence data are available in Table S24.

## Sequence typing

Serovars were predicted using the SeqSero2 version 1.1.1 (*Salmonella* Serotyping by Whole Genome Sequencing)^5^ and SISTR version 4.0.0 (*Salmonella* in silico typing resource)^6^. In addition to serotyping, seven conserved housekeeping genes (*aroC*, *dnaN*, *hemD*, *hisD*, *purE*, *sucA*, and *thrA*) were extracted from assembled contigs by multilocus sequence typing (MLST version 2.0) to determine the sequence types (STs)^7^. *Salmonella* genome sequences with unknown STs were submitted to Enterobase (http://enterobase.warwick.ac.uk/) and assigned new STs.

## Mapping and phylogenetic analysis

For analysis of SNPs, we use following genomes as reference genomes for SNP analysis, including *S*. Typhi str. CT18, *S*. Enteritidis str. P125109, *S*. Typhimurium str. LT2, *S*. Paratyphi B str. SPB7, *S*. Choleraesuis str. SC-B67 and *S*. Heidelberg str. SL476 using BWA version 0.7.17 with minimum mapping quality equal to 30 as default as previously described^8,9^. Candidate SNPs were identified and filtered using SAMtools version 1.10 which is a software for parsing and manipulating alignments in the generic aliment format (SAM/BAM format) as previously described^10^. The identified SNPs are converted into variant call format (VCF) using GATK version 4.1.4^11^ and summarized into a VCF file using BCFtools version 1.10^12^. Maximum-likelihood (ML) phylogenetic trees were built from SNP alignments using FastTree version 2.1.1^13^. Alleles from *S*. Paratyphi A str. AKU1_12601 were also included using the same mapping approach as an outgroup for phylogenetic tree rooting. To explore the correlation of *S*. I 1,4,[5],12:i:- ST34 strains between Chinese and other countries, 96 publicly available genomes^14,15^ were included in this study (Table S25). The interactive tree of life (iTOL version 6.1.1, https://itol.embl.de/) was used for phylogenetic tree visualization and annotation^16^. Tree tips were marked using strain name, source, year, provinces, STs, serovars, and country. The minimum spanning tree of multi-locus STs was generated in PHYLOViZ 2.0 (http://www.phyloviz.net/)^17^.

## Detection of antimicrobial resistance and virulence gene profiles and MGEs

The presence of acquired antimicrobial resistance genes and antimicrobial resistance associated with genomic point mutations were identified using ResFinder version 4.0^18^ (coverage ≥90% and identity ≥95%) and PointFinder databases (coverage =100% and identity ≥80%, accessed at January 28, 2021)^19^, respectively. Virulence genes were identified using ABRicate version 0.9.7 (<https://github.com/tseemann/abricate>) against the latest version of the Virulence Factor Database (VFDB)^20^ with threshold values of ≥80% identity and ≥80% coverage. *Salmonella* Pathogenic Islands (SPIs) were identified using SPIFinder version 2.0 (identity ≥95%, coverage ≥60%)^21^. Due to antimicrobial resistance genes (ARGs) are often associated with MGEs, including Miniature Inverted Repeats transposable elements (MITEs), Insertion sequences (ISs), Composite transposons (ComTns), Unit transposons (Tns), Integrative Conjugative Elements (ICEs), Cis-Mobilizable Elements (CIMEs) and Integrative Mobilizable Elements (IMEs), which can promote ARGs’ mobility, so MGEs were also investigated using MobileElementFinder version 1.0.3 (identity ≥90%, coverage ≥90%) hosted by the Center for Genomic Epidemiology (https://cge.cbs.dtu.dk/services/MobileElementFinder/)^22^. Plasmid types were identified using PlasmidFinder version 2.0.1 (identity ≥80%)^23^. PHASTER (http://phaster.ca/) was used to predict phage-like regions in high-quality assembled contigs^24^. The presence of *mcr-1*, *mcr-3*, *mcr-9*, and *tet*(X4) genes in *Salmonella* genomes was confirmed using Polymerase Chain Reaction (PCR) assays using primers in previous reports^25-28^ and 2 × Phanta Master Mix (Code: P511-03, Vazyme Biotech) and Sanger sequencing.

## Statistical analyses and visualization

Statistical significance was taken at *P* < 0.05. Multiple comparisons were performed by the Kruskal-Wallis test and Mann Whitney U test (unpaired t-test) using GraphPad Prism version 8.0. The descriptive analysis results are mainly shown as percentages. Stack columns, bar, and pie charts were generated using GraphPad Prism version 8.0. Venn diagrams were drawn in Venn Diagrams (http://bioinformatics.psb.ugent.be/webtools/Venn/). Heatmaps were plotted in the R platform with the pheatmap package. Region Map (http://bzdt.ch.mnr.gov.cn/) was used to map the geographical distribution of *Salmonella* isolates in China. The Easyﬁg (http://easyfig.sourceforge.net/)^29^ was used to visualize the genetic context comparisons.

## Data availability

All assembled Illumina sequence data are available from the National Center for Biotechnology Information under the BioProject number: PRJNA766315. Sequence reads generated for this study have been submitted to the National Microbiology Data Center (NMDC) under BioProject number: NMDC10017893 and NMDC10018145. The databases used for this study included MLST (https://bitbucket.org/genomicepidemiology/mlst_db/src/master/), SeqSero2 (https://github.com/denglab/SeqSero2), SISTR (https://github.com/phac-nml/sistr_cmd), VFDB (http://www.mgc.ac.cn/VFs/download.htm), PlasmidFinder (https://bitbucket.org/genomicepidemiology/plasmidfinder_db/src/master/), ResFinder (https://bitbucket.org/genomicepidemiology/resfinder_db/src/master/), PointFinder (https://bitbucket.org/genomicepidemiology/pointfinder_db/src/master/), SPIFinder (https://bitbucket.org/genomicepidemiology/spifinder_db/src/master/), and prophage (http://phaster.ca/databases), and MGE (<https://bitbucket.org/mhkj/mge_finder/src/master/>). The following reference genomes including *S*. Typhi str. CT18 (Accession no. AL513382), *S*. Enteritidis str. P125109 (BioSample: SAMEA1705941), *S*. Typhimurium str. LT2 (GenBank: AE006468), *S*. Paratyphi B str. SPB7 (GenBank: CP000886), *S*. Paratyphi A str. AKU_12601 (Accession no. FM200053), *S*. Choleraesuis str. SC-B67 (GenBank: AE017220), and *S*. Heidelberg str. SL476 (GenBank: CP001120) were downloaded from National Center for Biotechnology Information.

# Reference

1 Xu X, Chen Y, Pan H, et al. Genomic characterization of *Salmonella* Uzaramo for human invasive infection. *Microb Genom* 2020; **6**(7): mgen000401.

2 Bolger AM, Lohse M, Usadel B. Trimmomatic: a flexible trimmer for Illumina sequence data. *Bioinformatics* 2014; **30**(15): 2114-20.

3 Wick RR, Judd LM, Gorrie CL, et al. Unicycler: resolving bacterial genome assemblies from short and long sequencing reads. *PLoS Computat Biol* 2017; **13**(6): e1005595.

4 Seemann T. Prokka: rapid prokaryotic genome annotation. *Bioinformatics* 2014; **30**(14): 2068-9.

5 Zhang S, den Bakker HC, Li S, et al. SeqSero2: rapid and improved *Salmonella* serotype determination using whole-genome sequencing data. *Appl Environ Microbiol* 2019; **85**(23): e01746-19.

6 Yoshida CE, Kruczkiewicz P, Laing CR, et al. The *Salmonella* in silico typing resource (SISTR): an open web-accessible tool for rapidly typing and subtyping draft *Salmonella* genome assemblies. *PloS One* 2016; **11**(1): e0147101.

7 Larsen MV, Cosentino S, Rasmussen S, et al. Multilocus sequence typing of total-genome-sequenced bacteria. *J Clin Microbiol* 2012; **50**(4): 1355-61.

8 Li H, Durbin R. Fast and accurate short read alignment with Burrows-Wheeler transform. *Bioinformatics* 2009; **25**(14): 1754-60.

9 Ashton PM, Owen SV, Kaindama L, et al. Public health surveillance in the UK revolutionises our understanding of the invasive *Salmonella* Typhimurium epidemic in Africa. *Genome Med* 2017; **9**(1): 92.

10 Wong VK, Baker S, Pickard DJ, et al. Phylogeographical analysis of the dominant multidrug-resistant H58 clade of *Salmonella* Typhi identifies inter- and intracontinental transmission events. *Nat Genet* 2015; **47**(6): 632-9.

11 McKenna A, Hanna M, Banks E, et al. The genome analysis toolkit: a MapReduce framework for analyzing next-generation DNA sequencing data. *Genome Res* 2010; **20**(9): 1297-303.

12 Danecek P, McCarthy SA. BCFtools/csq: haplotype-aware variant consequences. *Bioinformatics* 2017; **33**(13): 2037-9.

13 Price MN, Dehal PS, Arkin AP. FastTree: computing large minimum evolution trees with profiles instead of a distance matrix. *Mol Biol Evol* 2009; **26**(7): 1641-50.

14 Ingle DJ, Ambrose RL, Baines SL, et al. Evolutionary dynamics of multidrug resistant *Salmonella* *enterica* serovar 4,[5],12:i:- in Australia. *Nat Commun* 2021; 12: 4786: 1-13.

15 Arai N, Sekizuka T, Tamamura-Andoh Y, et al. Identification of a recently dominant sublineage in *Salmonella* 4,[5],12:i:- sequence type 34 isolated from food animals in Japan. *Front Microbiol* 2021; 12: 690947.

16 Letunic I, Bork P. Interactive Tree Of Life (iTOL) v4: recent updates and new developments. *Nucleic Acids Res* 2019; **47**(W1): W256-W9.

17 Nascimento M, Sousa A, Ramirez M, et al. PHYLOViZ 2.0: providing scalable data integration and visualization for multiple phylogenetic inference methods. *Bioinformatics* 2017; 33: 128-129.

18 Bortolaia V, Kaas R, Ruppe E, et al. ResFinder 4.0 for predictions of phenotypes from genotypes. *J Antimicrob Chemother* 2020; 75: 3491-3500.

19 Zankari E, Allesoe R, Joensen KG, et al. PointFinder: a novel web tool for WGS-based detection of antimicrobial resistance associated with chromosomal point mutations in bacterial pathogens. *J Antimicrob Chemother* 2017; **72**(10): 2764-8.

20 Liu B, Zheng D, Jin Q, et al. VFDB 2019: a comparative pathogenomic platform with an interactive web interface. *Nucleic Acids Res* 2019; **47**(D1): D687-D92.

21 Roer L, Hendriksen RS, Leekitcharoenphon P, et al. Is the evolution of *Salmonella* *enterica* subsp. *enterica* linked to restriction-modification systems? *mSystems* 2016; **1**(3) :e00009-16.

22 Johansson MHK, Bortolaia V, Tansirichaiya S, et al. Detection of mobile genetic elements associated with antibiotic resistance in *Salmonella* *enterica* using a newly developed web tool: MobileElementFinder. *J Antimicrob Chemother* 2021; **76**(1): 101-9.

23 Carattoli A, Zankari E, Garcia-Fernandez A, et al. In silico detection and typing of plasmids using PlasmidFinder and plasmid multilocus sequence typing. *Antimicrob Agents Chemother* 2014; **58**(7): 3895-903.

24 Arndt D, Grant JR, Marcu A, et al. PHASTER: a better, faster version of the PHAST phage search tool. *Nucleic Acids Res* 2016; **44**(W1): W16-W21.

25 Liu YY, Wang Y, Walsh TR, et al. Emergence of plasmid-mediated colistin resistance mechanism MCR-1 in animals and human beings in China: a microbiological and molecular biological study. *Lancet Infect Dis* 2016; **16**(2): 161-8.

26 He T, Wang R, Liu D, et al. Emergence of plasmid-mediated high-level tigecycline resistance genes in animals and humans. *Nat Microbiol* 2019; 4(9): 1450-6.

27 Yin W, Li H, Shen Y, et al. Novel plasmid-mediated colistin resistance gene *mcr-3* in *Escherichia* *coli*. *mBio* 2017; **8**(3): e00543-17.

28 Carroll LM, Gaballa A, Guldimann C, et al. Identification of novel mobilized colistin resistance gene *mcr-9* in a multidrug-resistant, colistin-susceptible *Salmonella* *enterica* serotype Typhimurium isolate. *mBio* 2019; **10**(3): e00853-19.

29 Sullivan MJ, Petty NK, Beatson SA. Easyfig: a genome comparison visualizer. *Bioinformatics* 2011; **27**(7): 1009-10.
